# Contextualised strong reciprocity explains selfless cooperation despite selfish intuitions and weak social heuristics

**DOI:** 10.1038/s41598-021-93412-4

**Published:** 2021-07-06

**Authors:** Ozan Isler, Simon Gächter, A. John Maule, Chris Starmer

**Affiliations:** 1 School of Economics and Finance, Queensland University of Technology, Brisbane, 4000 Australia; 2 Centre for Behavioural Economics, Society and Technology, Brisbane, 4000 Australia; 3 School of Economics, University of Nottingham, Nottingham, NG7 2RD UK; 4 CESifo, 81679 Munich, Germany; 5 IZA, 53113 Bonn, Germany; 6 Leeds University Business School, University of Leeds, Leeds, LS6 1AN UK

**Keywords:** Psychology, Human behaviour

## Abstract

Humans frequently cooperate for collective benefit, even in one-shot social dilemmas. This provides a challenge for theories of cooperation. Two views focus on intuitions but offer conflicting explanations. The Social Heuristics Hypothesis argues that people with selfish preferences rely on cooperative intuitions and predicts that deliberation reduces cooperation. The Self-Control Account emphasizes control over selfish intuitions and is consistent with strong reciprocity—a preference for conditional cooperation in one-shot dilemmas. Here, we reconcile these explanations with each other as well as with strong reciprocity. We study one-shot cooperation across two main dilemma contexts, provision and maintenance, and show that cooperation is higher in provision than maintenance. Using time-limit manipulations, we experimentally study the cognitive processes underlying this robust result. Supporting the Self-Control Account, people are intuitively selfish in maintenance, with deliberation increasing cooperation. In contrast, consistent with the Social Heuristics Hypothesis, deliberation tends to increase the likelihood of free-riding in provision. Contextual differences between maintenance and provision are observed across additional measures: reaction time patterns of cooperation; social dilemma understanding; perceptions of social appropriateness; beliefs about others’ cooperation; and cooperation preferences. Despite these dilemma-specific asymmetries, we show that preferences, coupled with beliefs, successfully predict the high levels of cooperation in both maintenance and provision dilemmas. While the effects of intuitions are context-dependent and small, the widespread preference for strong reciprocity is the primary driver of one-shot cooperation. We advance the Contextualised Strong Reciprocity account as a unifying framework and consider its implications for research and policy.

## Introduction

Humans are an exceptionally cooperative species, willing to cooperate at personal cost in one-shot encounters with unrelated and even anonymous individuals^1^. Understanding cooperation in one-shot situations poses an important theoretical challenge from an evolutionary theory point of view^1–5^ because none of the mechanisms known to support cooperation among self-interested people, such as repeated interactions and reputation, are available in these circumstances^4,5^. An early explanation for this capacity was strong reciprocity^1,6–9^, a widespread cooperative preference reflecting inequality aversion^10,11^, concern for social efficiency^12^, and in particular, a desire to respond in kind to the perceived intentions of others^13,14^. Strong reciprocity differs from weak reciprocity, where cooperation can be rationalized by strategic thinking and selfish incentives^6^. In contrast, strong reciprocity amounts to a willingness to pay a personal cost for cooperating on the expectation that others do the same^7,15^.

The Social Heuristic Hypothesis (SHH) proposed a cognitive process explanation for cooperation in one-shot public good games that does not rely on strong reciprocity but on the distinction between intuition and deliberation^16–18^. Accordingly, self-interested people who benefit from cooperation in repeated interactions develop prosocial intuitions, which save cognitive effort at the risk of misapplying them in unfamiliar one-shot situations^18^. In the one-shot social dilemmas studied here, SHH therefore predicts that deliberation will tend to lower cooperation as people realise that cooperation does not pay^18^. However, the theoretical explanation of this effect is contested^19,20^, in particular because SHH conceives one-shot cooperation as a spill-over from weak reciprocity^21^ and thereby neglects the importance of strong reciprocity preferences for one-shot cooperation.

SHH has primarily been studied in provision dilemmas, where cooperation requires generosity to create or supplement a public good (e.g., donating blood, charitable giving, team work, collective action), and where spontaneous reactions tend to be cooperative^16,17,22,23^, though the extent to which this is the case has been challenged by more recent research^24–26^. However, cooperation often requires maintaining—that is, not exploiting—an already existing public good (e.g., clean environment, antibiotic efficacy, as well as civil society). Evidence from experiments comparing the provision and maintenance dilemmas suggests that ‘giving’ and ‘taking’ involve different psychological mechanisms^27–29^. Specifically, maintenance does not require generosity but rather restraint^27^ of selfish impulses to exploit the existing public good. Moreover, differences in the cognition of giving and taking can affect cooperation behaviour in provision and maintenance dilemmas through their influence on reciprocal motivations^28^. Cooperativeness has been shown to be weaker in maintenance than in provision dilemmas^30^. This raises the possibility that different cognitive processes, consistent with the Self-Control Account (SCA)^26,31–36^, underlie behaviour in the maintenance dilemma. According to SCA^31^ as applied to cooperation in social dilemmas^33,34^, people experience a selfish impulse to exploit the public good but can overcome this through the exercise of willpower to achieve cooperation. SCA therefore predicts that deliberation will increase cooperation by promoting resistance to selfish intuitions and enabling behaviour according to strong reciprocity preferences.

In this paper, we provide a comprehensive account of one-shot cooperation by combining the viewpoints of SHH and SCA that rely on the distinction between intuition and deliberation, with the preference-based theory of strong reciprocity. We use a general working definition of intuition that includes not only socially acquired heuristics (as in SHH) but also spontaneous emotional reactions (as in SCA). This is because the two cognitive process accounts posit different types of intuitions: while SHH emphasizes past social interactions in generating cooperative heuristics, SCA focuses on the role of visceral impulses for self-protection. In contrast, the strong reciprocity perspective does not consider the role of intuitions but assumes social preferences: it posits that cooperation reflects a motivation to reciprocate the cooperation expected from others. In addition to experimental manipulations involving time-limits, we provide analyses based on reaction times, social dilemma understanding, perceptions of social appropriateness, and beliefs about and preferences for cooperation.

Our formalization of the strong reciprocity explanation of one-shot cooperation predicts that one-shot cooperation is explained not only directionally but also quantitatively by a combination of preferences and beliefs. Incorporating SHH and SCH, our framework allows for the possibility that context-dependent (i.e., dilemma-specific) intuitions and perceptions affect preferences and beliefs. Consequently, we propose the Contextualised Strong Reciprocity (CSR) framework, which we support by experimental evidence across varying decision-making contexts. To achieve comparability across these contexts, we abstract from a variety of naturally occurring institutional and technological features^37–46^, and study intuitions and strong reciprocity in two fundamental types of social dilemmas: the provision and maintenance of public goods.

## Results

### Design

To experimentally compare maintenance and provision dilemmas, we used two formally equivalent and incentivized^47,48^ one-shot public goods games played in groups of four (*N* = 3653). In provision (P), each group member has a monetary endowment of 10 tokens which they can keep for themselves or invest in the public good. In maintenance (M), 40 tokens are already invested in the public good and each group member can withdraw up to 10 tokens for themselves. Selfish incentives are to contribute nothing in P or to withdraw 10 in M, but collective benefits are maximized by contributing 10 in P or by withdrawing nothing in M. We use incentivized^23^ between-subjects time-pressure (TP) and time-delay (TD) manipulations that prompt participants to respond within or after a particular time-limit to induce, respectively, relatively more intuitive or deliberated decisions. In doing so, we follow the original^16^ and the majority of studies on SHH^24,49–51^, which eases comparisons with the literature. We ran two pre-registered studies, which differed in the strength of time-pressure applied on cooperation decisions. As part of these studies, we also elicited strong reciprocity measures (expectations and preferences for conditional cooperation) as well as associated cognitive process measures (response times, social dilemma understanding, perceptions of social appropriateness and cognitive reflection) to investigate cooperation in the two social dilemmas.

### Are intuitions selfish or cooperative?

#### Hypotheses on SHH and SCA

Predictions of SHH and SCA diverge regarding the role of deliberation because SHH assumes that people tend to have cooperative intuitions but selfish preferences while SCA is consistent with a model of selfish intuitions and cooperative preferences. Our first objective is to test the predictions of SHH in P and M dilemmas and compare these with alternative predictions based on SCA. Drawing on previous literature^30,52–55^, we conjectured that contributions to the public good (labelled C: contributions in P or what is not withdrawn in M) will be higher in P than in M (H_1_: C_P_ > C_M_). SHH predicts^16^ that intuitive decisions will be more cooperative than deliberated decisions (H_2_: C_TP_ > C_TD_). Assuming that people have selfish preferences, SHH further predicts^17^ that deliberation will lower cooperation when intuitions are cooperative (as we assume in P) more so than when intuitions are selfish (as we assume in M). We therefore conjectured that differences in contributions between P and M (ΔC) will be driven by intuitive decisions and will dissipate with deliberation (H_3_: ΔC_TP_ > ΔC_TD_).

As an alternative hypothesis (H_A_) to H_2_, and in contrast to SHH, SCA predicts deliberation to increase cooperation (H_A_: C_TP_ < C_TD_). Self-control should be particularly important in M^56,57^, where intuitions are expected to be more selfish; hence SCA also predicts H_3_. However, SCA suggests a different cognitive process, one where deliberation increases cooperation by promoting self-control.

#### Study 1 found higher cooperation in P than M but supported neither SHH nor SCA

Study 1 included a no time-limit benchmark (NTL) alongside two time-limit conditions (TL), 10 s TP and 10 s TD. Compliance with time-limits was high in both TD (88.9%) and TP (93.6%). Indicating a successful experimental manipulation of behaviour, response times (RT) were faster in TP (mean, *M* = 6.15 s) than in TD (*M* = 28.17 s), *t*(1382) = 15.59, *P* < 0.001, *d* = 0.84.

**Figure 1:**
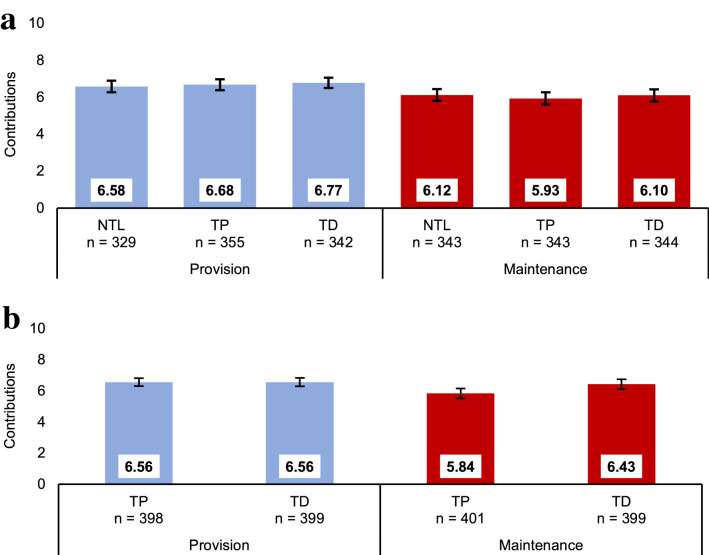
Cooperation in provision and maintenance dilemmas by time-limit conditions. Average cooperation (i.e., tokens contributed to or left in the public good, out of an endowment of 10) for provision (P) and maintenance (M) dilemmas. Numbers on bars are levels of cooperation. (**a**) Study 1: no time-limit (NTL), 10 s time-pressure (TP) and 10 s time-delay (TD) conditions. (**b**) Study 2: 5 s time-pressure (TP) and 10 s time-delay (TD) conditions. Error bars show 95% confidence intervals.

We found robust evidence for the effect of dilemma type on cooperation in the NTL and the TL samples (H_1_: C_P_ > C_M_) but found neither a time-limit nor an interaction effect (Fig. 1a). In NTL, the average tokens contributed was 4.6 percentage points (pp) higher in P (*M* = 6.58) than in M (*M* = 6.12), *t*(670) = 1.99, *P* = 0.048, *d* = 0.15. In TL, a two-way ANOVA showed 7.1 pp difference between P (*M* = 6.73) and M (*M* = 6.01), *F*(1, 1380) = 20.34, *P* < 0.001, η_p_^2^ = 0.015, but no difference in cooperation between TP (*M* = 6.31) and TD (*M* = 6.43) (H_2_), *F*(1, 1380) = 0.68, *P* = 0.408, η_p_^2^ < 0.001; we also found no interaction effect (H_3_), *F*(1, 1380) = 0.05, *P* = 0.825, η_p_^2^ < 0.001.

Survey questions on the extent to which decisions were based on deliberated vs. intuitive processes suggest that the 10 s time-limit was not short enough to disable deliberation relative to NTL (see Manipulation Checks in "Methods"). We therefore ran a second study using a stronger time-limit manipulation, with 5 s TP and 10 s TD.

#### Study 2 supported SCA—intuitions are selfish and more so in M than in P

In Study 2, manipulation checks supported the interpretation that decisions were made more intuitively in TP than in TD. The 5 s TP lowered RTs (*M* = 5.17 s) as compared to the 10 s TP condition in Study 1 (*M* = 6.15 s), *t*(1495) = 3.00, *P* = 0.003, *d* = 0.16. RTs were faster in TP (*M* = 5.17 s) than in TD (*M* = 28.45 s), *t*(1595) = 23.91, *P* < 0.001, *d* = 1.20 (see "Methods" for additional checks).

A two-way ANOVA indicated significant main effects of dilemma type and time-limits on cooperation (Fig. 1b). Supporting H_1_ (C_P_ > C_M_) and consistent with Study 1, contributions were higher in P (*M* = 6.56) than in M (*M* = 6.14), *F*(1, 1593) = 8.41, *P* = 0.004, η_p_^2^ = 0.005. However, the direction of the effect of time-limits on contributions (*M*_*TP*_ = 6.20 vs. *M*_*TD*_ = 6.50) was the opposite to that predicted by SHH (H_2_: C_TP_ > C_TD_) and in line with SCA’s prediction of selfish intuitions and increased cooperation with deliberation (H_A_: C_TP_ < C_TD_), *F*(1, 1593) = 4.09, *P* = 0.043, η_p_^2^ = 0.003.

The interaction between time-limit and dilemma type was also significant, indicating that intuitions are context-dependent. As predicted (H_3_: ΔC_TP_ > ΔC_TD_), the difference in contributions between the two dilemmas was higher under TP (ΔC_TP_ = 7.2 pp) than under TD (ΔC_TD_ = 1.3 pp), *F*(1, 1593) = 3.99, *P* = 0.046, η_p_^2^ = 0.002. This was because average contributions in M were significantly lower under TP than under TD, *t*(798) = 2.63, *P* = 0.009, *d* = 0.19, whereas no effect could be identified in P, *t*(795) = 0.02, *P* = 0.985, *d* < 0.01.

#### Study 2 showed limited evidence for SHH in P

Finally, we compared the effect of time-limits on the prevalence of zero contributions in P and complete withdrawal in M because some interpretations of SHH make predictions about the likelihood of free-riding behavior rather than the extent of cooperation^18^. While this exploratory analysis provided no further insights to the null results in Study 1 (the prevalence of zero contributions in P being 3.1% in TP vs. 2.0% in TD and in M being 10.5% in TP vs. 10.5% in TD), we found support in Study 2 for SHH in P (prevalence of zero contributions being 0.8% in TP vs. 4.8% in TD) but not in M (11.2% in TP vs. 8.3% in TD). Specifically, a logit model of free-riding behaviour in Study 2, χ^2^(3, *n* = 1597) = 50.37, *P* < 0.001, indicated a significant interaction between dilemma type and time-limits (*P* < 0.001) such that time delay increased the likelihood of zero contributions in P (*OR* = 6.58, *P* = 0.003) but not in M (*OR* = 0.71, *P* = 0.161).

In short, confirmatory tests supported SCA’s prediction that time-delay increases cooperation, an effect that was stronger in M than in P. In contrast, we only found limited, exploratory evidence based on measures of free-riding for SHH’s predictions in P. Overall, the effect of intuitions on contributions depended on the social dilemma type but tended to be small (*d* < 0.20). These findings suggest that cognitive processes driving cooperation differ across the P and M dilemmas. Next, we explore this difference further by investigating the relationship between cooperation decisions and response times, which are considered indicative of the extent of deliberation underpinning a decision^58,59^.

#### Fast decisions are more selfish in M than in P

**Figure 2:**
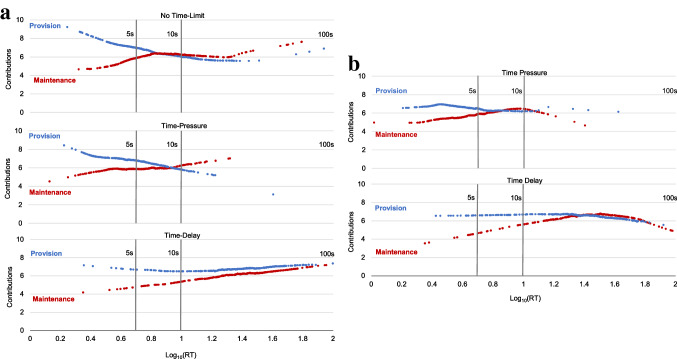
Cooperation by response times. (**a**) Study 1: Plotted are cooperation and response times (RT) in NTL, TP and TD. (**b**) Study 2: Plotted are cooperation and RT in TP and TD. The graphs show LOWESS estimates, representing the relationship between tokens contributed to the public good and RTs, shown for each social dilemma. Each series is composed of OLS estimates in the locality of each RT observation (bandwidth 0.8). Estimates for three responses (0.2%) that took longer than 100 s are not shown. Corresponding linear model estimates are in Tables S1 and S2.

We log-transform RTs (to base 10) to account for data skewness^16^ and depict the relationship between RT and cooperation across the time-limit conditions using local nonparametric estimates of contributions over time. Visual comparison of the two dilemmas in both Study 1 (Fig. 2a) and Study 2 (Fig. 2b) suggests that fast decisions, when compared to slow decisions, tend to be more prosocial in P and more selfish in M. Comparing the two dilemmas, contributions seem to be more prosocial in P and more selfish in M especially among fast decisions. The corresponding linear model estimates verify these visual trends (Tables S1 & S2). Considering the two studies together, contributions in P were higher than in M by 13.5 pp for decisions made within the first 5 s, *t*(1017) = 7.54, *P* < 0.001, *d* = 0.47; by 3.8 pp for decisions made in 5 s to 10 s, *t*(1048) = 2.01, *P* = 0.044, *d* = 0.12; and by 1.4 pp for decisions longer than 10 s, *t*(1582) = 0.92, *P* = 0.357, *d* = 0.05.

Two alternative interpretations have been offered for the RT analysis of cooperation decisions: the earlier argument that fast RTs indicate intuition^16,58,59^ and the more recent view that they reflect lack of conflict during decision-making due to strong preference for one option over others^60^. While it may be difficult to disentangle these two influences in a correlational analysis^60,61^, our RT analysis is consistent with the experimental findings in Study 2 of intuitive selfishness in M and modest deliberated free-riding in P. Moreover, we do not find clear evidence for decision conflict accounts predicting that faster decisions are more extreme (contributing nothing or everything)^62^ or predicting that TP increases random decision error^61,63,64^ or arguing for congruency of choices with preferences^65,66^ (see Supplementary Materials for details, including a revised decision conflict account model consistent with our data).

Whether driven by decision conflict or dual-process mechanisms^67^, the differences in RTs suggest systematic differences in the cognitive processes underlying behaviour in the two dilemmas. Next, we present converging exploratory evidence that supports this view, showing dilemma-dependent differences in (1) understanding of social dilemmas, (2) perceptions of social appropriateness, and (3) reliance on deliberative rather than intuitive thinking.

### Intuitions are consistent with other cognitive processes underlying cooperation

#### M promotes self-gain understanding, P promotes group-gain understanding

An important question is how people understand the P and M dilemmas^64,68–70^. From a standard game-theoretic point of view, M and P are strategically identical: contributing nothing and withdrawing everything maximize self-gain, contributing everything and withdrawing nothing maximize group welfare. We measured participants’ correct understanding of these strategies using two incentivized questions (Methods).

**Figure 3:**
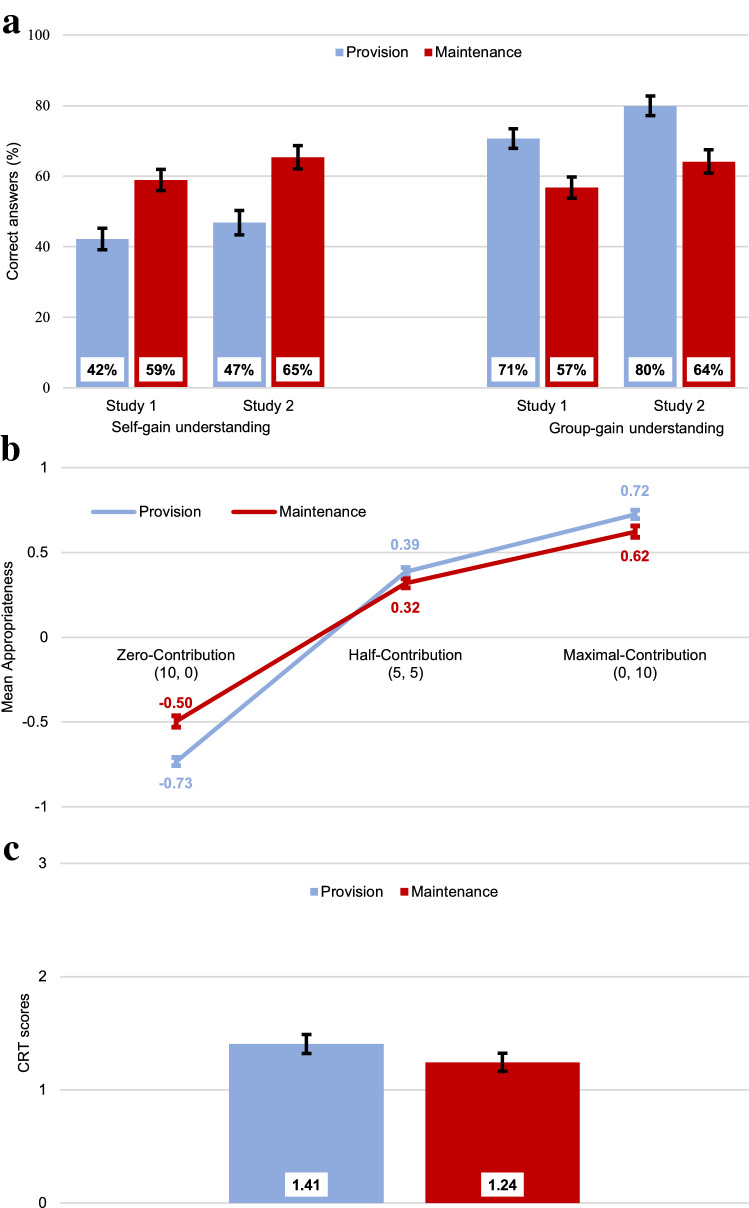
Dilemma-specific differences in cognitive processes. (**a**) Percentage of people who correctly answered the two questions on understanding about the self-gain maximization strategy and the group-gain maximization strategy. (**b**) Perceptions of social appropriateness of three decision scenarios: contributions of 0, 5 and 10 (or withdrawals or 10, 5 and 0), corresponding to “zero-contribution”, “half-contribution” and “maximal-contribution” (only elicited in Study 1). **c**, Number of correct answers on the Cognitive Reflection Test (only elicited in Study 2). Error bars show 95% confidence intervals. See "Methods" for detailed definition of measures and Supplementary Materials for additional analyses.

In both studies, the correct understanding of the self-gain maximizing strategy was higher in M (61.8% overall) than in P (44.2%), whereas understanding of the group-gain strategy was higher in P (74.7%) than in M (60.0%) (Figs. 3a, S1); χ^2^-tests, *P*s < 0.001. Across the two dilemmas, those who correctly understood the self-gain maximizing strategy tended to be less cooperative (*M* = 6.13) than those who misunderstood it (*M* = 6.61), *t*(3651) = 4.96, *P* < 0.001, *d* = 0.16, while those who correctly understood the group-gain maximizing strategy tended to be more cooperative (*M* = 6.95) than those who misunderstood it (*M* = 5.14), *t*(3651) = 18.02, *P* < 0.001, *d* = 0.64.

These contrasting effects of the two components of social dilemma understanding tended to cancel each other when we used—like related literature^16^—a composite understanding variable that equated one if both self- and group-gain questions were correctly answered and zero otherwise. Specifically, consistent with our preregistered two-way ANOVA model of Study 2, the effects of social dilemma type (*P* = 0.003), time-limits (*P* = 0.049) and their interaction (*P* = 0.046) were significant in an exploratory model that controlled for social dilemma understanding. In a less conventional second exploratory model that took correct answers to the two understanding questions separately, neither social dilemma type (*P* = 0.326) nor time-limits (*P* = 0.171) were significant. Nevertheless, consistent with the confirmatory evidence found for SCA in Study 2, the increase in contributions in TD over TP in M was similar for those with (5.1 pp) and without understanding (5.9 pp).

#### Freeriding is more socially appropriate in M than P

Perceptions of social appropriateness can guide cooperation decisions^71,72^. Employing a standard approach that involves an incentivized coordination game^71^, participants in Study 1 estimated how socially appropriate other participants perceived various levels of contribution in P or withdrawal in M (Methods). Because our public good games were anonymous and one-shot, these perceptions are independent of the characteristics and actions of one’s group members. Cooperation was negatively correlated with the perceived appropriateness of contributing nothing (*r*_*s*_ = − 0.25, *P* < 0.001) or half of the endowment (*r*_*s*_ = − 0.35, *P* < 0.001) and positively correlated with the perceived appropriateness of maximal contributions (*r*_*s*_ = 0.28, *P* < 0.001). Zero-contribution was perceived to be *less* socially appropriate in P than in M (Fig. 3b), *t*(2054) = 11.09, *P* < 0.001, *d* = 0.49. In contrast, half-contribution was perceived to be more socially appropriate in P than in M; *t*(2054) = 3.77, *P* < 0.001, *d* = 0.17. Likewise, social appropriateness of maximal-contribution was higher in P than in M, *t*(2054) = 4.78, *P* < 0.001, *d* = 0.21.

#### M increases reliance on intuitions more than P

The Cognitive Reflection Test^73^ (CRT) was elicited at the end of Study 2 to test whether treatment effects depended on individual thinking styles (Methods). As expected, there was neither an effect of time limits on CRT scores—the total number of correct answers—nor an interaction between time-limits and dilemma types. However, CRT scores were significantly higher in P than in M (Fig. 3c), *F*(1, 1593) = 7.32, *P* = 0.007, η_p_^2^ = 0.005.

The dilemma-specific difference in CRT scores is surprising because the allocation of participants to P and M was random. The probability that this asymmetry was due to sampling bias is low (< 5%) as confirmed by simulations that randomly assigned each observed CRT score to one of two hypothetical experimental conditions and tested for difference in average scores (see Supplementary Materials). We also tested for sampling bias by re-inviting all Study 2 participants one-and-a-half years later to take part in a supplementary study that measured their CRT scores for a second time; as planned, we stopped data collection when we reached 50% of the original sample (*n* = 800). Among these re-invited participants, the initial CRT score was 9.7% higher for those who had experienced P than those who were in M, χ^2^(3) = 9.95, *P* = 0.019. In contrast, when we compared their second CRT scores, we found no difference, χ^2^(3) = 2.71, *P* = 0.439.

An alternative scoring of the CRT is to measure the total number of intuitive but incorrect answers (iCRT^74^; Methods). Consistent with standard scoring, exposure to M resulted in 7.6% higher iCRT than P, χ^2^(3) = 8.67, *P* = 0.034, yet this difference was no longer evident the second time iCRT was measured, χ^2^(3) = 1.42, *P* = 0.701. These exploratory findings, which require replication in future research, suggest that dilemma type may influence thinking styles. In particular, exposure to M may decrease reliance on reflection possibly by triggering stronger and more enduring intuitive reactions as compared to P.

These measures provide convergent evidence of systematic differences in the cognitive processes underpinning P and M dilemmas that are consistent with the differences in intuitions observed earlier. Overall, cooperation was higher in P than in M in all five time-manipulation conditions across the two studies (Fig. 1). Nevertheless, in contrast to SHH’s assumption that preferences are selfish and its prediction that deliberation erodes cooperation, we found substantial levels of cooperation in the time-delay conditions across the two studies (67% of the endowment was contributed in P and 63% in M). We next test whether these high levels of cooperation can be explained by strong reciprocity^1,6^.

### Strong reciprocity explains one-shot cooperation

#### Measures of strong reciprocity

The key evidence for strong reciprocity is the preference for conditional cooperation despite net personal costs in anonymous one-shot social dilemmas^9,15^. We elicited, in both studies, two incentivized measures of conditional cooperation—expectations and preferences^75^. First, expectations are point estimates of the average expected contribution level of other group members (Methods). Expectations (here also referred to as expected cooperation) can be indicative of strong reciprocity motives in anonymous one-shot games because they are independent of how other participants actually behave^75–80^. Second, individual preferences were measured by eliciting contribution schedules (i.e., a public good contribution decision for each possible average contribution level, from 0 to 10 tokens, of others in their group)^75^. Based on pre-registered criteria about the relationship between own and other contributions, we classified each participant as conditional cooperator (strong positive correlation), free rider (zero contributions regardless of others’ contributions) or other^75^. Our protocol also provides a predicted contribution for each individual, found by combining their contribution schedule with their expectations (Methods).

#### Hypotheses on strong reciprocity

We test three preregistered hypotheses on dilemma-specific differences in strong reciprocity based on previous evidence^30^: more people will be conditional cooperators (CC) in P than M (H_4_: CC_P_ > CC_M_); expectations (E) will be higher in P than M (H_5_: E_P_ > E_M_); and predicted contributions (PC) will be higher in P than in M (H_6_: PC_P_ > PC_M_). Next, we present results of tests that combine data from the two studies (see "Methods" for additional hypotheses and Supplementary Materials for detailed results).

#### Strong reciprocity is weaker in M than in P

**Figure 4:**
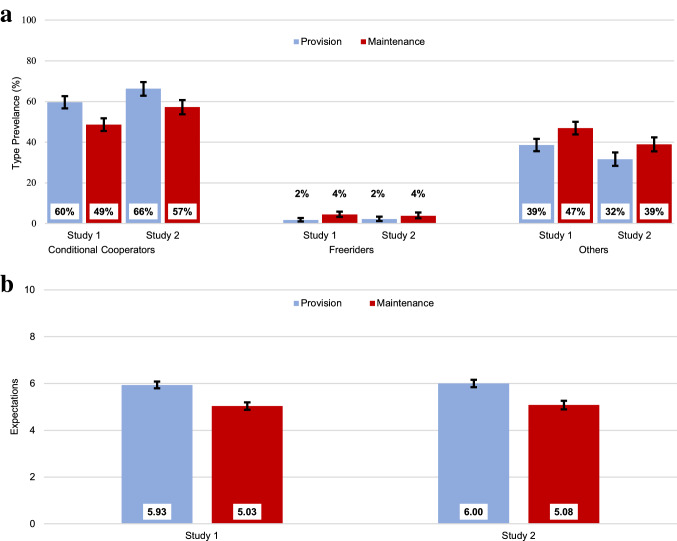
Strong reciprocity measures. (**a**) Distribution of social preference types: conditional cooperators (left panel), free riders (middle panel) and other social preference types (right panel). (**b**) Expectations about the average contribution made by others in the group. Error bars show 95% confidence intervals. See "Methods" for definition of expectation measures and preference type categorization.

As hypothesized (H_4_), the frequency of conditional cooperators was significantly higher in P (63%) than in M (52%), χ^2^(1, *n* = 3653) = 38.34, *P* < 0.001, whereas preference for free-riding, though surprisingly rare overall^81^, was more prevalent in M (4%) than in P (2%), χ^2^(1, *n* = 3653) = 16.08, *P* < 0.001 (Fig. 4a). Across both studies, and consistent with H_5_, expectations about others’ contributions were significantly higher in P (*M* = 5.96) than in M (*M* = 5.05), *t*(3651) = 11.03, *P* < 0.001, *d* = 0.36 (see Figs. 4b and S2). Although weaker, predicted contributions were also on average higher in P (*M* = 5.76) than M (*M* = 5.56), *t*(3651) = 1.95, *P* = 0.051, *d* = 0.06 (H_6_). Expectations were elicited using the same time-limits that participants faced during contribution decisions. However, we found no effect of time-limits on expectations or predicted contributions. The lack of effect of time-limits on strong reciprocity measures is consistent with the view that motivation for strong reciprocity is equally high for intuitive and deliberated decisions. Next, we explore whether the dilemma-specific differences in strong reciprocity are related to how people cognitively process these two social dilemmas.

#### Understanding, social appropriateness, and reflection influence strong reciprocity

Linear regressions across the two studies (see Table S4) indicated that expectations regarding group members’ contributions depended on social dilemma understanding (increasing with group-gain and decreasing with self-gain understanding) as well as on perceptions of social appropriateness (increasing with the perceived appropriateness of maximal contributions and inappropriateness of zero- and half-contributions; all *P*s < 0.001). In addition, logit models of cooperation preferences showed that the likelihood of being categorized as a conditional cooperator increased with social dilemma understanding, with the tendency for reflective thinking and with perceived appropriateness of cooperation. Hence, these measures of cognitive processes were consistently associated with measures that indicate motivation for strong reciprocity.

#### Strong reciprocity explains cooperation in both dilemmas

Irrespective of the dilemma-specific differences (Figs. 1, 2, 3), cooperation was high (> 60%) in both dilemmas (*M*_*P*_ = 6.63 and *M*_*M*_ = 6.09). These high levels of cooperation need an explanation that SHH cannot provide. SHH predicts lower cooperation in TD than TP because deliberation should increase the saliency of free-riding. In fact, cooperation tended to increase slightly with time-delay across the two studies (*M*_*TP*_ = 6.25 vs *M*_*TD*_ = 6.47), *t*(2979) = 2.01, *P* = 0.045, *d* = 0.07. SHH also implies that cooperation should decrease with social dilemma understanding^18^, experience with one-shot social dilemma games^82^, and the Cognitive Reflection Test score^17^. However, all three variables showed either significantly positive or insignificant correlations with cooperation (Table S3).

**Figure 5:**
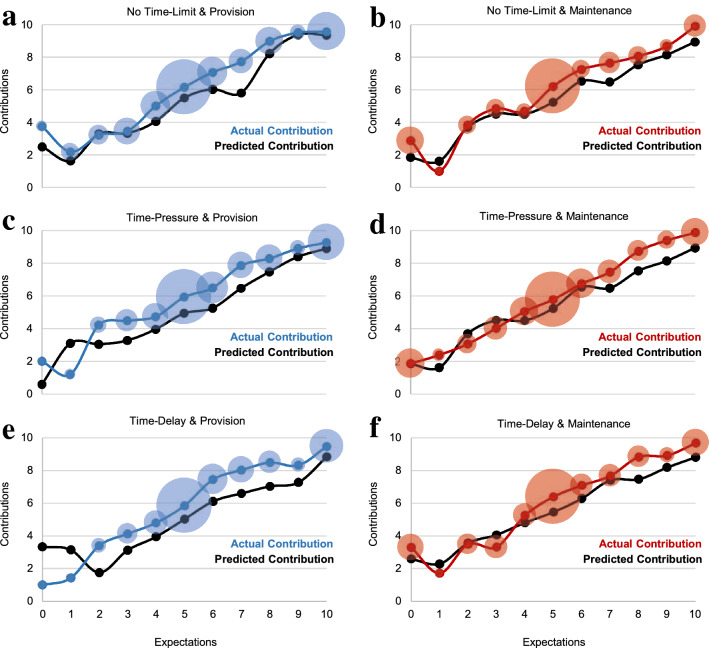
Actual and predicted contributions by expectations across experimental conditions. Coloured lines show average actual contribution at each expectation level reported by participants. The size of the bubble indicates the number of participants at that particular expectation level. The black lines denote average predicted contribution found by combining expectation with the deliberated contribution preference schedule (Methods). (**a, b**) Study 1 only; (**c**–**f**) Study 1 and 2 pooled. (**a**) Participants under no time-limit (NTL) in provision dilemma (P). (**b**) Participants under NTL in maintenance dilemma (M). (**c**) Participants under time-pressure (TP) in P. (**d**) Participants under TP in M. (**e**) Participants under time-delay (TD) in P. (**f**) Participants under TD in M. Figures S4 and S5 present this analysis separately for those with (Fig. S4) and without (Fig. S5) correct social dilemma understanding, with very similar results.

The high cooperation levels in our data can instead be explained by strong reciprocity. Specifically, actual contributions were highly correlated with expectations (*r*_*s*_ = 0.65, *P* < 0.001) and predicted contributions (*r*_*s*_ = 0.54, *P* < 0.001) (Fig. 5). Expectations and predicted contributions remained strongly positively correlated with contributions when we controlled for the covariates of social dilemma understanding, experience and CRT score (Table S3). The correlations between expected and actual contributions were strongly positive for those with (*r*_*s*_ = 0.63) and without (*r*_*s*_ = 0.66) correct social dilemma understanding (Figs. S4, S5) and not only for conditional cooperators (*r*_*s*_ = 0.65) but also for free riders (*r*_*s*_ = 0.68) and those classified as “others” (*r*_*s*_ = 0.62; all *P*s < 0.001) (Fig. S6).

**Figure 6:**
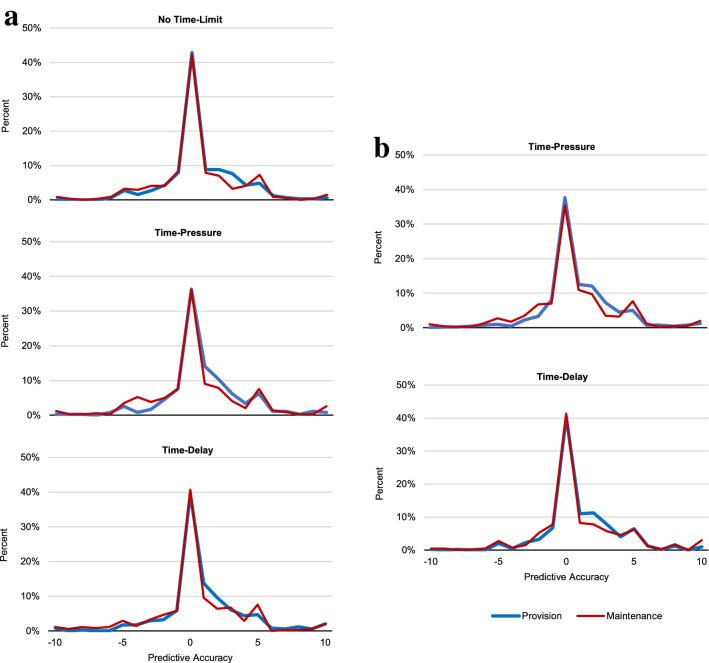
Accuracy of strong reciprocity measures in predicting cooperation. (**a**) Study 1: Distribution of predictive accuracy (the difference between actual and predicted contributions for each participant) in NTL, TP and TD. (**b**) Study 2: Distribution of predictive accuracy in TP and TD. Note that zero indicates highest predictive accuracy possible. Figures S7 and S8 present this analysis separately for those with (Fig. S7) and without (Fig. S8) correct social dilemma understanding, with very similar results.

Strong reciprocity explains both aggregate and individual cooperation. At the aggregate level, predicted contributions accounted for 87% of actual contributions in P and 91% of actual contributions in M. At the individual level, as preregistered, we categorized those with predicted contributions within 10% (i.e., ± 1 token) of their actual contributions as exhibiting predictive accuracy^83^ (Methods). In both studies, 57% of participants showed predictive accuracy at the 10% level, and only 6% of all participants showed predictive inaccuracy by more than 50% (i.e., ± 6 or more tokens) (Fig. 6). The consistency of predicted contributions did not depend on dilemma type, time-limit manipulations or their interaction, since the corresponding logit models of predictive accuracy were overall insignificant in both Study 1 (*P* = 0.479) and Study 2 (*P* = 0.452).

### Contextualised Strong Reciprocity (CSR) account of one-shot cooperation

Our results indicate contextualised (i.e., dilemma-dependent) strong reciprocity preferences explain anonymous one-shot cooperation. Our account has three key components. First, the context-specific features of the social dilemma (e.g., dilemma type) systematically shape intuitions as reflected in associated cognitive process measures (Figs. 2, 3). Second, expectations of others’ cooperation and preferences for strong reciprocity are also influenced by the decision context (Fig. 4). Third, the prevalence of motivations for strong reciprocity explains the high levels of cooperation observed in one-shot anonymous social dilemmas (Figs. 5, 6).

**Figure 7:**
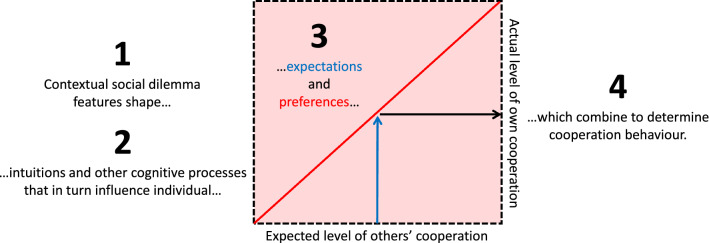
The Contextualised Strong Reciprocity (CSR) account of one-shot cooperation. CSR suggests four steps for explaining why people cooperate in anonymous one-shot social dilemmas: (1) The individual faces a specific decision context involving a social dilemma (e.g., Provision or Maintenance), which (2) shapes intuitions (e.g., social heuristics or selfish impulses) and other cognitive processes (e.g., influencing understanding of incentives, perceptions of social appropriateness of cooperation and the tendency for cognitive reflection). These in turn influence (3) expected cooperation by others (the horizontal axis represents the range of expectations and, for example, the blue arrow represents the expectation that others will contribute half of their endowments) as well as individual preferences for cooperation (the pink area represents the range of possible preferences and, for example, the red line represents the preferences of a perfect conditional cooperator). (4) Actual cooperation behaviour is a function of an individual’s preferences, and in the case of strong reciprocity, also a function of the expected level of cooperation by others (as exemplified by the black arrow). In our studies, evidence for the relationship between steps 1 and 2 is in Figs. 2 and 3; evidence for the relationship between steps 2 and 3 is in Fig. 4; and evidence for the relationship between steps 3 and 4 is in Figs. 5 and 6.

We combine these components in the CSR account, a unified framework for understanding why and how people cooperate in one-shot social dilemmas (Fig. 7). Formally put, CSR explains cooperation as $a_{i} \left( {f,~b_{i} \left( f \right)} \right) \to c_{i}$, where an individual *i*’s contribution level $(c_{i} )$ is a function of *i*’s preference for conditional cooperation $(a_{i} )$ and *i*’s expectation about other’s cooperation $(b_{i} )$. Both $a_{i}$ and $b_{i}$ are functions of the contextual features $\left( f \right)$ of a given social dilemma (see Fig. 4). CSR posits that $a_{i} \left( {f,~b_{i} \left( f \right)} \right)|f~$ predicts $c_{i}$ equally well for all *f*—that is the prediction error, $c_{i}  - c_{i}^{*}$, is equally distributed across *f*—a proposition that is supported by our data (Fig. 6). As discussed in the next section, CSR extends beyond the specifics of the games studied here, serving as a general framework for understanding cooperation in one-shot social dilemmas.

## Discussion

In two experiments, we studied the psychological mechanisms underlying cooperation across maintenance and provision dilemmas. Our results provide converging evidence that, despite their formal equivalence, P and M dilemmas trigger systematic differences in cooperation behaviour and its underlying psychological processes. Cooperation is higher in P than in M (Fig. 1). We show that this gap is consistent with dilemma-specific intuitions, which are more selfishly oriented in M than in P (Figs. 2, 3). Measures of strong reciprocity (Fig. 4), being weaker in M than P, also depend to some extent on intuitions. Nevertheless, cooperation in both dilemmas remains high even with deliberation and is well-predicted by preferences for strong reciprocity (Figs. 5, 6).

Our time-limit manipulations allowed systematic comparison of the two main cognitive process accounts of cooperation: SHH which predicts intuitive cooperation and SCA which predicts intuitive selfishness. While deliberation increases free-riding in P, we found that it promotes cooperation in M. Hence, SHH’s prediction of intuitive cooperation does not extend to M dilemmas, where SCA’s prediction of intuitive selfishness applies. CSR integrates the contrasting mechanisms postulated in SHH and SCA by combining effects driven by different types of intuitions: SHH invokes social heuristics that tend to promote cooperation, whereas SCA invokes visceral, emotional reactions that tend to promote self-regard. Our study suggests that the relative importance of either type of intuition can depend systematically on contextual factors such as the social dilemma type, but further research on CSR is needed to understand the nature of these (and other) types of intuitions and the context-specific cues that trigger them. As these results are exclusively based on time-pressure manipulations (with methodological drawbacks such as high levels of non-compliance), their robustness should be tested using alternative, stronger cognitive process manipulations in future research^84–87^.

CSR explains why finding evidence for SHH has proven difficult. Recent meta-analyses of the literature on SHH indicate a small effect size of promoting intuitive thinking on cooperation or no effect at all^24,49–51,88^. One reason may be the coexistence of two independent and opposing intuitions in social dilemmas such that the negative effect of selfish intuitions (as argued by SCA) tends to cancel out the positive effect of social intuitions on cooperation (as argued by SHH)^26^. Another reason may simply be that intuitions tend to have relatively small effects on cooperation, especially relative to the role that strong reciprocity plays in one-shot or ‘selfless’ cooperation.

Indeed, the preference for strong reciprocity is prevalent not only in our data (Fig. 4a) but across numerous studies^81,89,90^. We observed high levels of deliberated cooperation, with time-delayed contributions being 63% in M and 67% in P (Fig. 1). Consistent with CSR’s formulation of $a_{i} \left( {f,~b_{i} \left( f \right)} \right)$, the prevalence of strong reciprocity preferences and high levels of expectations jointly predicted this behavioural outcome well, with predicted contributions of 56% in M and 57% in P for the TD conditions (Fig. 5). The high levels of deliberated cooperation motivated by strong reciprocity restricts the range for observing even higher cooperation due to social heuristics (e.g., under TP) to 37 pp in M and 33 pp in P. Isolating differences in cooperation levels supporting SHH in the P dilemma would therefore rely on intuitions being significantly more cooperative on average than this already high level. In this sense, a sample of individuals with relatively more selfish preferences (i.e., with lower deliberated cooperation) and with previous exposure to repeated cooperative interactions would be more likely to provide evidence for SHH.

The potential applicability of CSR extends beyond the P and M dilemmas studied here. The contextual features $\left( f \right)$ of any particular cooperation problem (e.g., social dilemma type) will trigger *f*-specific intuitions and strong reciprocity preferences. In combination, these provide a general framework to explain cooperation in any one-shot social dilemma.

At this early stage, CSR is theoretically mute on the potentially complex relationship between *f* and the specific preferences, intuitions, and other cognitive processes that it triggers. Instead, CSR is primarily a framework for explaining one-shot cooperation that, for a given *f*, emphasizes measurement of expectations and preferences. As part of this empirical analysis, auxiliary measures of cognitive processes provide insights into how the specific *f* of a social dilemma situation influences cooperation. For example, our measurements (Figs. 2, 3) suggest that intuitions and other cognitive processes are more selfishly oriented when *f* pertains to M than to P. This is consistent with CSR’s theoretical prediction that preferences and expectations jointly explain cooperation and our empirical observation that behaviour is selfishly biased in M compared to P (Fig. 4). While expectations and preferences constitute the primary determinants of cooperation in CSR, intuitions and auxiliary measures are particularly relevant when comparing cooperation across contexts, when seeking insights into the cognitive processes underlying cooperation and when formulating frames for the effective delivery of public policies.

The stark cognitive and behavioural differences between P and M dilemmas show that researchers and policymakers should not presume that the formal equivalence of the two dilemmas on standard theoretical analysis implies behavioural equivalence and should instead distinguish between provision and maintenance problems both analytically and for policy purposes. The generalizability of our results to naturally occurring environments should be tested in field studies^91^, for example, in attempts to maintain environmental public goods. Serious global threats such as excessive energy consumption or antibiotic overuse involve maintenance problems, where selfish impulses threaten the collective interest. In these cases, our results indicate that policymakers should engage individuals in deliberation^57^ to promote collective welfare. Crucially, as our CSR account shows, policymakers should primarily strive to harness the power of strong reciprocity to motivate cooperation.

## Methods

### Experimental design

We ran two experiments designed to investigate intuitions and strong reciprocity across the two main social dilemma types (i.e., the Provision and Maintenance dilemmas). Each study had four main parts. Part A elicited decisions in one-shot public good games (PGGs) with time-limits, and Parts B to D elicited various additional cognitive process measures. The methods of the two studies were common except in the few aspects detailed below. Both studies employed between-subject designs crossing dilemma type (P or M) by time-limit (Study 1: 10 s pressure, 10 s delay or no time-limit; Study 2: 5 s pressure or 10 s delay). We adapted the P and M dilemmas developed for lab-based research^30^ for an online setting, and employed standard time-limit manipulations^16^ and incentivized compliance with time-limits^23^. Simple randomization was used, and participants were blind to the experimental conditions. Both studies were preregistered at the Open Science Framework (https://osf.io/euw9d/).

### Ethics

Our research complies with all relevant ethical regulations. Ethics approval was obtained from the University of Nottingham School of Economics Research Ethics Committee, and informed consent was obtained from the participants.

### Participants

We recruited participants via Prolific (www.prolific.ac)^92^, restricted to native English-speaking UK residents who were 18 years or older. Study 1 participants were not allowed to participate in Study 2. We use data from 2,056 participants in Study 1 (age: *M* = 36.6, *SD* = 11.4; female: 75.2%) and from 1,597 participants in Study 2 (age: *M* = 34.2, *SD* = 11.6; female: 65.0%), excluding 153 incomplete submissions across the two studies. Because experience with (or other knowledge of) public goods experiments may weaken the effect of time-limit manipulations^82^, we recruited participants from Prolific, where most participants reported no prior knowledge or experience with PGGs (89.9% in Study 1 and 89.2% in Study 2).

### Stakes

Participants were paid according to their decisions^47,48^. Including a participation fee of £0.50, average earning was £1.43 in Study 1 and £1.38 in Study 2 with a median completion time of 8 min in Study 1 and 10 min in Study 2. This corresponds to a fee of approximately £11 per hour, which is considerably higher than the minimum wage in the UK (about £8 at the time of the experiments).

### Planned sample size

#### Study 1

In a one-shot PGG, previous research^30^ observed higher cooperation in P than in M dilemma with an effect size (*d*) of 0.59. In a pilot study (*n* = 92), we observed a smaller effect in the same direction (*d* = 0.28). A related study on time-pressure^23^ found time-pressured decisions to be more cooperative than time-delayed decisions (*d* = 0.22). We used the smallest of the three effects as our benchmark and calculated the sample size required to detect an effect in a two-tailed *t*-test to be at least *n* = 338 per treatment group (*α* = 0.05, 1-*β* = 0.80). Considering possible incomplete submissions, we aimed to recruit 2100 participants.

#### Study 2

In a pilot study (*n* = 91), we established the feasibility of using a 5 s time-limit by comparing compliance in 4 s (27.3%), 5 s (64.3%) and 6 s (70.0%) TP limit conditions. Motivated by previous research^93^, we used the effect size of the interaction term in the Study 1 sample from an OLS model of PGG contributions on dilemma type, binary response time (RT) variable (1 if RT ≥ 5 s), and their interaction (f = 0.0815). With a more powerful test than in Study 1 (α = 0.05, 1-β = 0.90), the sample size required to detect an interaction effect between time-manipulation and dilemma type in an ANOVA was found to be at least *n* = 396 per treatment group. Considering possible incomplete submissions, we aimed to recruit 1600 participants.

### Randomization

Data on P and M dilemmas were simultaneously collected and Qualtrics survey software (www.qualtrics.com) was used to randomly allocate participants to treatments. Lack of significant differences in the distribution of the number of participants (χ^2^-tests, *P* = 0.780 for Study 1 and *P* = 0.940 for Study 2) across the experimental conditions in both studies as well as for demographic variables such as gender (χ^2^-tests, *P* = 0.577 for Study 1 and *P* = 0.385 for Study 2) and age (Kruskal–Wallis tests, *P* = 0.106 for Study 1 and *P* = 0.243 for Study 2) indicate that simple randomization worked as intended.

### Procedures

We programmed the experiments using *Qualtrics*. Participants first received slider training (the mechanism used for recording participants’ responses) and PGG instructions for either the P or the M dilemma, and then completed four decision-making parts. In Part A, participants played a one-shot linear PGG. In Study 1, Part A was completed under 10 s time-pressure (TP), 10 s time-delay (TD) or no time-limit (NTL), whereas in Study 2, Part A was completed under 5 s TP or 10 s TD. In Part B, we first elicited expectations regarding other group members’ contributions (under TP, TD or NTL congruent with Part A) and then elicited understanding of the social dilemma. We elicited cooperation preferences in Part C. In Part D, we either elicited perceptions of social appropriateness^71^ for three scenarios (Study 1) or the three-item Cognitive Reflection Task (CRT)^73^ (Study 2). Finally, participants completed a brief demographic survey. Participants were informed at the beginning that one of the four parts would be randomly selected at the end of the study to calculate their additional earnings. We detail the components of the procedure below.

#### Slider training

Participants were familiarized with the slider tool—later used to elicit contribution decisions—on a practice screen^23^. To prevent anchoring, the tool did not have a default slider position. The training was intended to minimize differences in familiarity with the tool that would have otherwise occurred between time-pressured and other participants.

#### Public good game

We used a one-shot linear public good game with four-person groups. Each token kept (or withdrawn) earned this group member one token; each token contributed to (not withdrawn from) the public good returned half a token to each group member that is, two tokens for the group as a whole, constituting a social dilemma. Each group was randomly assigned to instructions for either the P or the M dilemma. The two tasks are equivalent in terms of the relationship between allocations to the public good and monetary earnings from the public good game^30^. We opted for the brief instructional style introduced by Rand et al.^16^ without control questions prior to the game rather than the extended analytical style standard in lab experiments^30^. This was intended to minimize inducement of a calculative mindset, which may weaken the capacity of time-limits to induce intuitive thinking^16^.

### Part A

#### Time manipulations and PGG decision

Participants were randomly assigned to a time-limit condition for their PGG decisions. In Study 1, we adopted the standard limits^16^—a 10 s threshold for both the TP and the TD conditions. In Study 2, we intensified the TP treatment by using a 5 s threshold while retaining a 10 s threshold for TD. The TP conditions prompted participants to “be quick” and decide “in less than 10 [5] seconds”, whereas the TD conditions prompted them to “carefully consider” their decision “for more than 10 s”. NTL did not use a time-limit prompt. To minimize noncompliance with time-limits, participants in the TP and TD conditions were initially informed of the upcoming time-limits and that noncompliant participants would be ineligible for earnings from the public good game^23^. This transitory screen was displayed for a fixed period of 15 s, which was long enough to allow reading and short enough to prevent deliberation about the upcoming task.

### Part B

#### Expectations

Participants next guessed the average number of tokens contributed to (P) or withdrawn from (M) the public good by the three other people in their group (i.e., expected cooperation). Congruent with the time-limit condition that was assigned during the public good decision, these expectations were elicited under either TP, TD or NTL, thus exactly mimicking the conditions of the public good game environment. If the task was selected for payment, participants were rewarded £0.50 for correct predictions that also complied with time conditions. Prior to the elicitation of beliefs, a transitory screen was displayed for a fixed period of 20 s describing the reward conditions. This screen was displayed for 5 s longer than the transitory screen prior to the public good decision to account for the slightly longer text. No additional time-limits were used in the study after this question.

#### Social dilemma understanding

Understanding of the social dilemma was measured by two questions displayed on the same screen in random order each asking participants to choose a contribution (withdrawal) level from 0 to 10 tokens to identify: the correct strategy for maximizing own monetary gain (no contribution in P or full withdrawal in M) and the correct strategy for maximizing group’s monetary gain (full contribution in P or no withdrawal in M). An initial screen informed the participants that the questions had correct answers each worth £0.50 and prompted those who previously were under time-limits to decide at their “own pace”. Following the literature^16^, we categorize a participant as having understood the social dilemma if both questions were answered correctly (41.0% in Study 1 and 47.4% in Study 2; these figures are comparable with previous studies such as 45.6% in Study 6 of Rand, Greene and Nowak, 2012, an online PGG experiment using time-limits).

### Part C

#### Measuring preferences for (conditional) cooperation

The preference elicitation task ^15,30,94^ involved a modified public good game, which asked participants to provide a contribution (P) or withdrawal (M) level *for each possible rounded average level of contribution or withdrawal* that could have been made by the other three people in the participant’s group. Using eleven sliders on the same screen—each corresponding to a possible scenario ranging from an average contribution of others of 0, 1, …, 10 tokens (randomly presented either in ascending or descending order)—the participants indicated their preferred contribution or withdrawal for each possible level of others’ average contributions (i.e., conditional cooperation). If Part C was selected for payment, payments were calculated by randomly selecting one group member for whom the conditional contribution at the average of others’ one-shot PGG contributions was payoff-relevant; for the others their initial (unconditional) PGG contributions were used to calculate payoffs. This is an incentive compatible method to elicit preferences for conditional cooperation^94^. Monetary costs and returns from contributing to the public good in this task were equivalent to those from the public good game described above.

The contribution profiles elicited in these tables are used to categorize each participant into one of three cooperation preference types^30^. Those who consistently contributed nothing to (or withdrew everything from) the public good in all eleven scenarios are categorized as “free riders” (3.1% in Study 1 and 3.0% in Study 2). Participants are categorized as “conditional cooperators” if the eleven contribution (withdrawal) decisions show a weakly monotonically increasing pattern for own contribution (withdrawal) in relation to others’ average contributions (withdrawals) or if the Spearman rank correlation coefficient between own and others’ contributions (withdrawals) is positive and significant at the 1% level (54.1% in Study 1 and 61.7% in Study 2). Remaining participants are categorized as “other” (42.8% in Study 1 and 35.3% in Study 2).

#### Predicted contributions

This measure^30^ provides a prediction of public good contribution for each participant by combining the individual’s cooperation preference and expected cooperation measures: using the expectations measure, we first find the scenario in the preference elicitation task that is expected by the participant to be true, and we then identify the contribution level stated for that scenario as the participant’s predicted contribution.

### Part D

#### Perceptions of social appropriateness

Social appropriateness was measured in Study 1 using an incentivized coordination game^71^ for three possible actions in the public good game: 0, 5 or 10 tokens contributed to (10, 5, 0 tokens withdrawn from) the public good. The three questions were randomly displayed on the same screen either in ascending or descending order. Using a six-point scale ranging from “very socially inappropriate” to “very socially appropriate”, participants were asked to estimate how socially appropriate most people in the study would find each action. Participants were informed that they would be randomly matched with someone in the study, that one of the three actions would be randomly chosen, and that if this task was chosen to calculate extra earnings then they would each earn £1 if the two evaluations matched. The task thus constitutes a coordination game that incentivizes honest reporting of participants’ perceptions of the social appropriateness of various actions in the public good game. Responses were converted to numerical scores^71^ so that 0 implies “neutral”: “very socially inappropriate” =  − 1, “somewhat socially inappropriate” =  − 2/3, “socially inappropriate” =  − 1/3, “socially appropriate” = 1/3, “somewhat socially appropriate” = 2/3, “very socially inappropriate” = 1.

#### Cognitive reflection test

We elicited the three-item CRT^73^ in standard order twice, first at the end of Study 2 and then one-and-a-half years later. If Part D in Study 2, which included the CRT, was selected for payment, then participants were rewarded £0.50 for completing the task. The second time the CRT was measured, we invited all Study 2 participants and continued data collection until 50% of participants were recruited. In this follow up experiment, CRT was measured in isolation, with no preceding play of P or M dilemma games. We calculated two types of CRT scores for each individual. The standard CRT scores (between 0 and 3) were calculated as the total number of correct answers. The alternative iCRT scores (also ranging from 0 to 3) were calculated as number of intuitive but incorrect answers (see Supplementary Materials for the CRT questions and further information)^74^.

### Hypotheses

#### Main hypotheses

We describe our main preregistered hypotheses in the main text. The first three hypotheses make predictions about contribution decisions. We predicted higher contributions (C) in P than in M (H_1_: C_P_ > C_M_). Consistent with SHH, we also predicted higher contributions in TP than in TD (H_2_: C_TP_ > C_TD_) and higher difference in contributions between P and M in TP than in TD (H_3_: ΔC_TP_ > ΔC_TD_). As an alternative to H_2_, SCA predicts higher contributions in TD than in TP (H_A_: C_TP_ < C_TD_). Our other three main hypotheses involve predictions about measures of strong reciprocity: conditional cooperators (CC) will be more prevalent in P than in M (H_4_: CC_P_ > CC_M_); beliefs about others’ contributions, expectations (E), will be higher in P than in M (H_5_: E_P_ > E_M_); and predicted contributions (PC) will be higher in P than in M (H_6_: PC_P_ > PC_M_).

#### Additional tests

Extending H_2_ (C_TP_ > C_TD_) to strong reciprocity measures, we tested the hypotheses of higher conditional cooperation (H_7_: CC_TP_ > CC_TD_), expectations (H_8_: E_TP_ > E_TD_) and predicted contributions (H_9_: PC_TP_ > PC_TD_) in TP than in TD. Similarly, we extend H_3_ (ΔC_TP_ > ΔC_TD_) to reciprocity measures (H_10_: ΔCC_TP_ > ΔCC_TD_, H_11_: ΔE_TP_ > ΔE_TD_, H_12_: ΔPC_TP_ > ΔPC_TD_). However, cooperation schedules were elicited without time-limits. H_7_ and H_10_ should therefore be viewed as testing spill-over of previous time-limits (applied during elicitation of C and E) on type elicitation. All twelve hypotheses were tested for both Study 1 and Study 2, and the results of additional hypotheses tests are reported in the Supplementary Materials.

Two additional hypotheses on the initial CRT scores were preregistered in Study 2. In particular, because contributions are expected to be higher in P than in M for intuitive decisions and because low CRT scores indicate tendency for intuitive thinking, we predicted that contributions in P will be higher than in M among those with a CRT score of zero (H_13_) and that the effect of dilemma type on contributions will be weaker for higher CRT scores (H_14_). Although we report these analyses for completeness in the Supplementary Materials, these cannot be interpreted as valid tests of H_13_ and H_14_ due to unexpected spill-over effects from dilemma type on subsequently elicited CRT scores.

### Analysis plan

We report tests for our main hypotheses in the Results section of the main text and introduce exploratory analyses as appropriate. We provide detailed results in the Supplementary Materials for the main and the additional hypothesis tests (see Hypotheses). Our preregistered tests do not control for any of the covariates sometimes used in the literature as control measures (i.e., age, gender, experience with or knowledge of the public good game, compliance with time-limits and social-dilemma understanding). The results in both studies are robust when controlling for these covariates.

We recode all withdrawal-based decisions in the M condition as tokens kept in the public good game, and we refer to these values as contributions to the public good. First, we present our preregistered test results. As planned, we analyse the no time-limit (NTL) and the time-limit samples (TL: TP & TD) separately. To test the first twelve hypotheses as appropriate, we use χ^2^ and t-tests in the NTL sample of Study 1, and we use two-way ANOVA and Logistic interaction models in the TL samples of Study 1 and Study 2. These models include the time-limit (TP vs. TD), the dilemma type (P vs. M) and their interaction term as covariates. The models differ by their dependent variable: contributions (C), expectations (E) or predicted contributions (PC) in ANOVAs and prevalence of conditional cooperators (CC) in Logistic regressions. To find main effects on CC in the TL samples, we use χ^2^ tests. Apart from ANOVAs and χ^2^ tests that are based on distributions with one tail, all tests are two-tailed.

### Manipulation checks

As our main manipulation check, in both studies we compared the differences in mean response times between the time-limit conditions. In addition to this behavioral measure reported in the main text, we elicited and compared self-reported cognitive manipulation checks.

#### Study 1

A post-experimental survey question asked participants to compare how intuitively vs. deliberatively they decided in the PGG on a 4-point scale ranging from very intuitively (1) to very deliberatively (4). The exact wording of the question depended on the social dilemma condition: “Please indicate the degree to which you decided intuitively vs. deliberatively while choosing how many tokens to contribute to [withdraw from] the group project.” Although the difference in answers between TP (*M* = 2.23) and TD (*M* = 2.32) was significant, t(1382) = 2.08, *P* = 0.037, *d* = 0.11, responses did not differ between NTL (*M* = 2.29) and either the TP or the TD conditions (NTL vs TP: t(1368) = 1.27, *P* = 0.205, *d* = 0.07; NTL vs TD: t(1356) = 0.75, *P* = 0.453, *d* = 0.04).

#### Study 2

This study introduced a cognitive manipulation check by eliciting two simple evaluations right after the measurement of expectations (i.e., the end of time-limits): (1) “I did not have time to think through my decisions” and (2) “I decided based on my ‘gut reaction’”. Ratings for the two statements ranged on a 5-point scale from “strongly disagree” (1) to “strongly agree” (5). The two questions were presented on the same screen in randomized order. Ratings of the first statement indicate that TP (*M* = 3.37) limited opportunities for deliberation compared to TD (*M* = 1.55), t(1595) = 32.28, *P* < 0.001, *d* = 1.62. Ratings of the second statement indicate that TP (*M* = 4.06) increased spontaneous decisions compared to TD (*M* = 3.76), t(1595) = 5.60, *P* < 0.001, *d* = 0.28.

### Compliance with time-limits

We include all non-compliant participants in the analyses, since exclusion may bias estimatesy^25^.

#### Study 1

Compliance with time-limits based on RTs was high in both TD (Overall: 88.9%; P: 89.8%; M: 88.1%), and TP (Overall: 93.6%; P: 94.9%; M: 92.1%).

#### Study 2

Due to increased time-pressure (5 s), the standard time-limit compliance rate based on the submission of the decision page (i.e., RT) was lower in TP (Overall: 58.9%; P: 63.6%; M: 54.4%) than in TD (Overall: 91.9%; P: 89.5%; M: 94.2%). Nevertheless, supporting measures indicate that participants in TP were strongly motivated to comply with the time limits. Specifically, the median RT among those who failed to comply with the TP limits was still relatively fast (6.4 s) such that more than a third of these participants (38.7%) submitted their responses within 5 to 6 s (i.e., within one additional second above the limit), nearly a quarter (22.5%) submitted their responses within 6 to 7 s, and overall, 87.5% of all noncompliant participants submitted their responses within 5 to 10 s.

### Statistical analysis

As preregistered, we conducted most of our analyses using ANOVAs and t-tests. Our main results hold when we use nonparametric tests. The statistical tests used are described in the main text, and the analysis codes will be available upon publication at the Open Science Framework project site.

## Supplementary Information

Supplementary Information.

## Data Availability

The datasets, materials and analysis codes are available at the Open Science Framework project site (https://osf.io/euw9d/).
