# Supplementary material for: Contextualised strong reciprocity explains selfless cooperation despite selfish intuitions and weak social heuristics
Source: Sci Rep. 2021 Jul 6;11:13868. doi: 10.1038/s41598-021-93412-4 (PMC8260766; doi:10.1038/s41598-021-93412-4)
Supplement: Supplementary file 1 — Supplementary Information. [file 41598_2021_93412_MOESM1_ESM.pdf]

Supplementary Information for

Contextualised strong reciprocity drives selfless cooperation despite selfish intuitions and weak social heuristics

Ozan Isler[1,2]*, Simon Gächter[3,4,5]*, A John Maule[6], Chris Starmer[3]

[1] School of Economics and Finance, Queensland University of Technology, Brisbane, Australia 4000
[2] Centre for Behavioural Economics, Society and Technology, Brisbane, Australia 4000
[3] School of Economics, University of Nottingham, Nottingham, UK NG7 2RD
[4] CESifo Munich, Germany 81679
[5] IZA Bonn, Germany 53113
[6] Leeds University Business School, University of Leeds, Leeds, UK LS6 1AN

* Corresponding authors: Ozan Isler; Simon Gächter.

Emails: ozan.isler@qut.edu.au; simon.gaechter@nottingham.ac.uk

8 June 2021

This PDF file includes:

Supplementary Figures S1 to S8
Supplementary Tables S1 to S4
Supplementary Results
Supplementary Methods
Supplementary References

1. Supplementary Figures

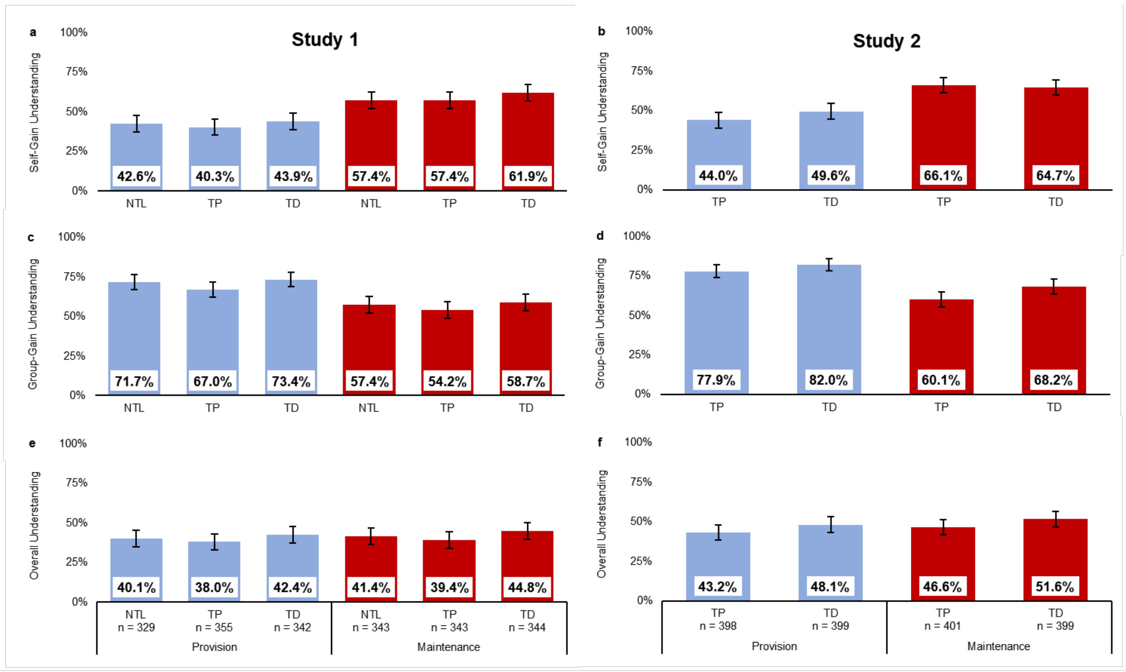

g Logit models of understanding (Study 1)

	Self-Gain (1)	Group-Gain (2)	Overall (3)
Dilemma (0 if P, 1 if M)	2.000*** (0.308)	0.582*** (0.091)	1.054 (0.166)
Time-Limit (0 if TP)			
NTL	1.098 (0.171)	1.247 (0.208)	1.092 (0.171)
TD	1.158 (0.178)	1.356* (0.226)	1.199 (0.186)
Dilemma*Time-Limit			
M & NTL	0.911 (0.200)	0.913 (0.207)	0.997 (0.220)
M & TD	1.040 (0.228)	0.886 (0.201)	1.041 (0.228)
Constant	0.675*** (0.073)	2.034*** (0.230)	0.614*** (0.067)
χ2 (1, n = 2056)	59.56	46.94	4.09
Prob > χ2	< .001	< .001	.536

h Logit models of understanding (Study 2)

	Self-Gain (1)	Group-Gain (2)	Overall (3)
Dilemma (0 if P, 1 if M)	2.483*** (0.363)	0.428*** (0.068)	1.148 (0.163)
Time-Limit (0 if TP, 1 if TD)	1.255 (0.179)	1.289 (0.229)	1.219 (0.174)
Dilemma*Time-Limit	0.748 (0.154)	1.103 (0.255)	1.002 (0.201)
Constant	0.785** (0.079)	3.523*** (0.426)	0.761*** (0.077)
χ2 (1, n = 1597)	57.75	55.78	5.82
Prob > χ2	< .001	< .001	.121

Fig. S1. Social dilemma understanding. a, c, e, g, Study 1 only. **b, d, f, h,** Study 2 only. **a, b,** Correct understanding of the self-gain maximization strategy (i.e., zero contribution or complete withdrawal, see Methods). **c, d,** Correct understanding of the group-gain maximization strategy (i.e., maximal contribution or zero withdrawal). **e, f,** Correct overall understanding of the social dilemma (i.e., correct understanding of both self-gain and group-gain maximization strategies) across experimental conditions. Error bars show 95% confidence intervals. **g, h,** Logit models of (self-gain, group-gain and overall) understanding on dilemma type, time-limit condition and their interaction. Experimental manipulations had no effect on overall social dilemma understanding in either study. In both studies, understanding of the self-gain maximizing strategy was better in M than in P, and the understanding of the group-gain strategy was better in P than in M, indicating asymmetries in game perceptions consistent with selfish intuitions in M and cooperative intuitions in P. Odds ratios are reported. Robust SE in parentheses. * $P < .10$, ** $P < .05$, *** $P < .01$.

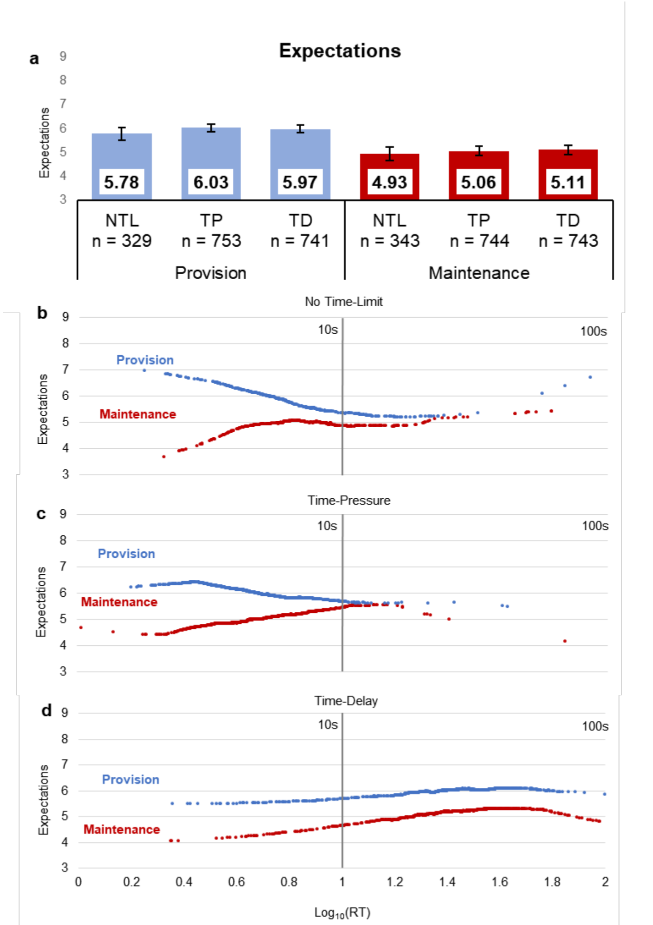

e

OLS models of expectations

	NTL (1)	TP (2)	TD (3)
Dilemma (0 if P, 1 if M)	-0.872***	-0.994***	-0.861***
	(0.199)	(0.128)	(0.128)
$\log_{10}$(RT)	-1.066	-1.201***	0.517*
	(0.671)	(0.444)	(0.300)
Dilemma*$\log_{10}$(RT)	1.363	2.321***	0.369
	(0.877)	(0.653)	(0.487)
Constant	5.790***	6.027***	5.993***
	(0.138)	(0.084)	(0.082)
	F(3, 668) = 7.36	F(3, 1493) = 25.72	F(3, 1480) = 19.54
Prob > F	< .001	0.008	< .001

Fig. S2. Expectations in provision and maintenance dilemmas by time-limit conditions (Study 1 & 2 pooled). a, Expected cooperation by the other group members (i.e., tokens contributed to or left in the public good, out of an endowment of 10), for provision (P) and maintenance (M) dilemmas in the no time-limit (NTL), time-pressure (TP) and time-delay (TD) conditions. Numbers on bars are average levels. Error bars show 95% confidence intervals. **b**, Expectation and response times (RT) in NTL. **c**, Expectation and RT in TP. **d**, Expectation and RT in TD. Line graphs show LOWESS estimates, which represent the relationship between expected tokens contributed to the public good and RTs, shown for each social dilemma, and composed of OLS estimates in the locality of each RT observation (bandwidth 0.8). Estimates for ten responses (0.3%) that took longer than 100s are not shown. **e**, OLS models of expected contributions on dilemma type and median-centered log of RT [log(RT)-log(*MD*)] estimated for each time-limit condition in tokens: no time-limit (NTL), time-pressure (TP) and time-delay (TD). Median RT is 6.6s in NTL, 4.8s in TP and 25.3s in TD. Robust SE in parentheses. * *P* < .10, ** *P* < .05, *** *P* < .01.

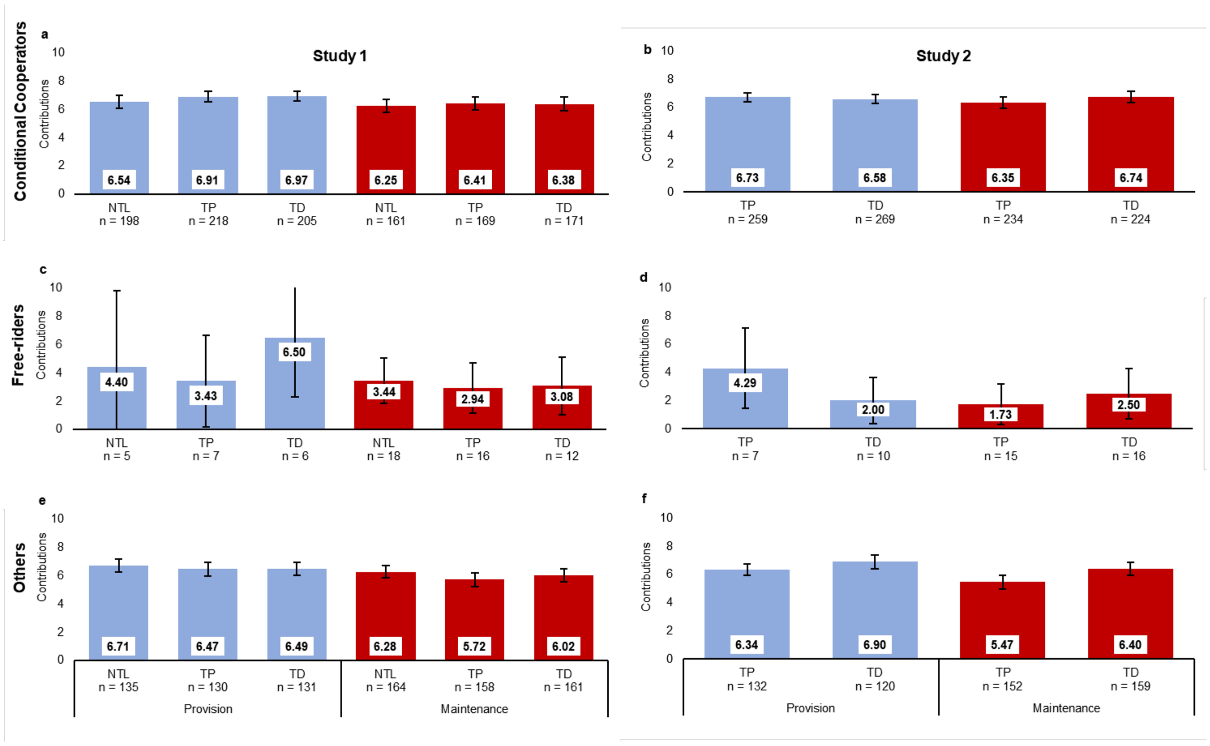

Fig. S3. Cooperation in provision and maintenance dilemmas by time-limit conditions for each cooperation preference type. Average cooperation (i.e., tokens contributed to or left in the public good, out of an endowment of 10) for provision (P) and maintenance (M) dilemmas. Numbers on bars are levels of cooperation. **a**, **b**, conditional cooperators; **c**, **d**, free-riders; **e**, **f**, others. **a**, **c**, **e**, Study 1: no time-limit (NTL), 10s time-pressure (TP) and 10s time-delay (TD) conditions. **b**, **d**, **f**, Study 2: 5s time-pressure (TP) and 10s time-delay (TD) conditions. Error bars show 95% confidence intervals.

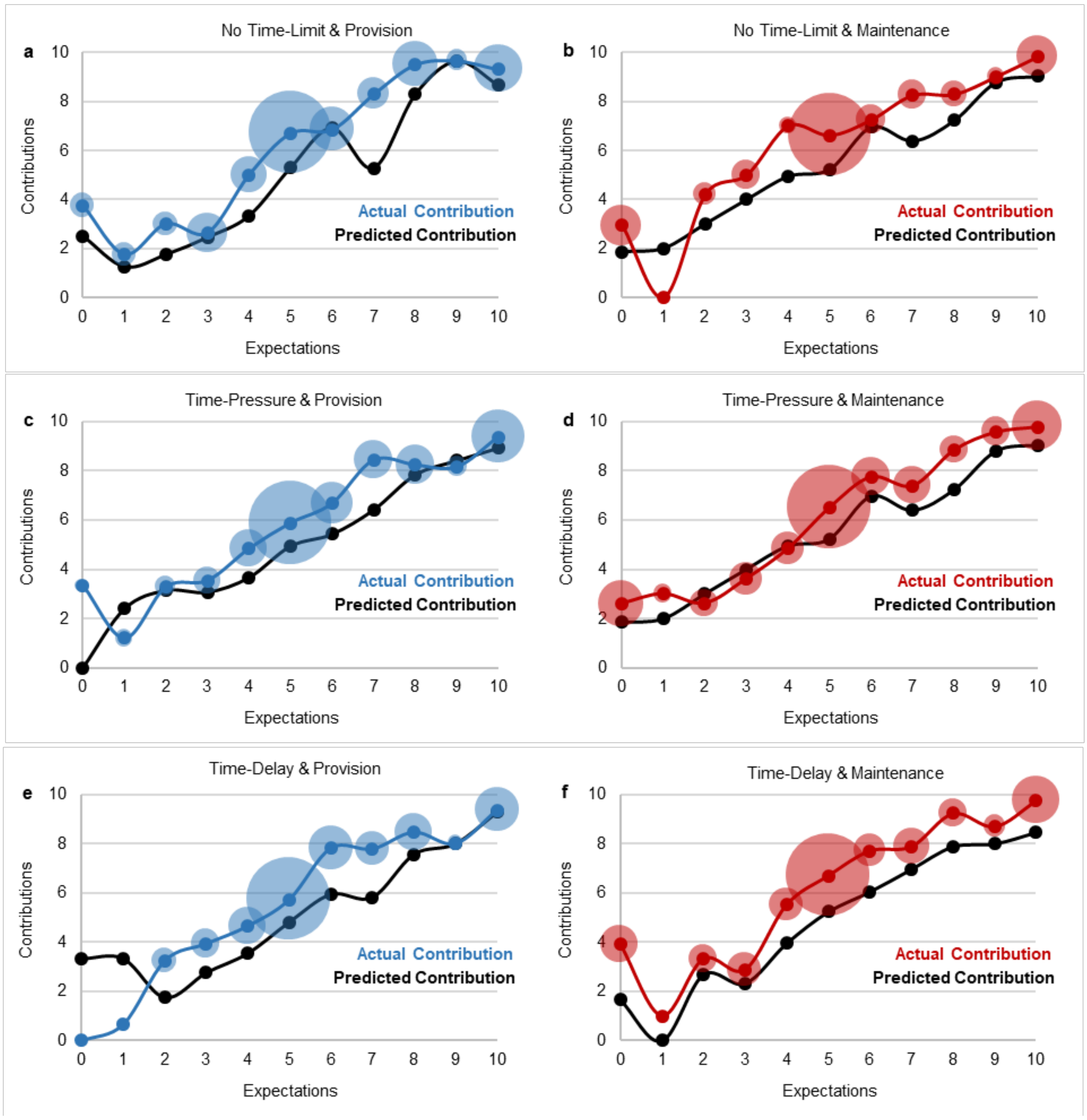

Fig. S4. Actual and predicted cooperation by expectations among participants with correct social dilemma understanding. a, **b**, Study 1 only; **c** – **f**, Study 1 and 2 pooled. Included are only participants who correctly answered the two understanding questions (i.e., the correct strategy for maximizing own monetary gain and for maximizing group monetary gain). **a**, Participants under no time-limit (NTL) in provision dilemma (P). **b**, Participants under NTL in maintenance dilemma (M). **c**, Participants under time-pressure (TP) in P. **d**, Participants under TP in M. **e**, Participants under time-delay (TD) in P. **f**, Participants under TD in M. Colored lines show average actual contribution at each expectation level reported by participants. The size of the bubble indicates the number of participants with respective expectation level. The black lines denote average predicted contribution found by combining expectation with the deliberated contribution preference schedule (Methods).

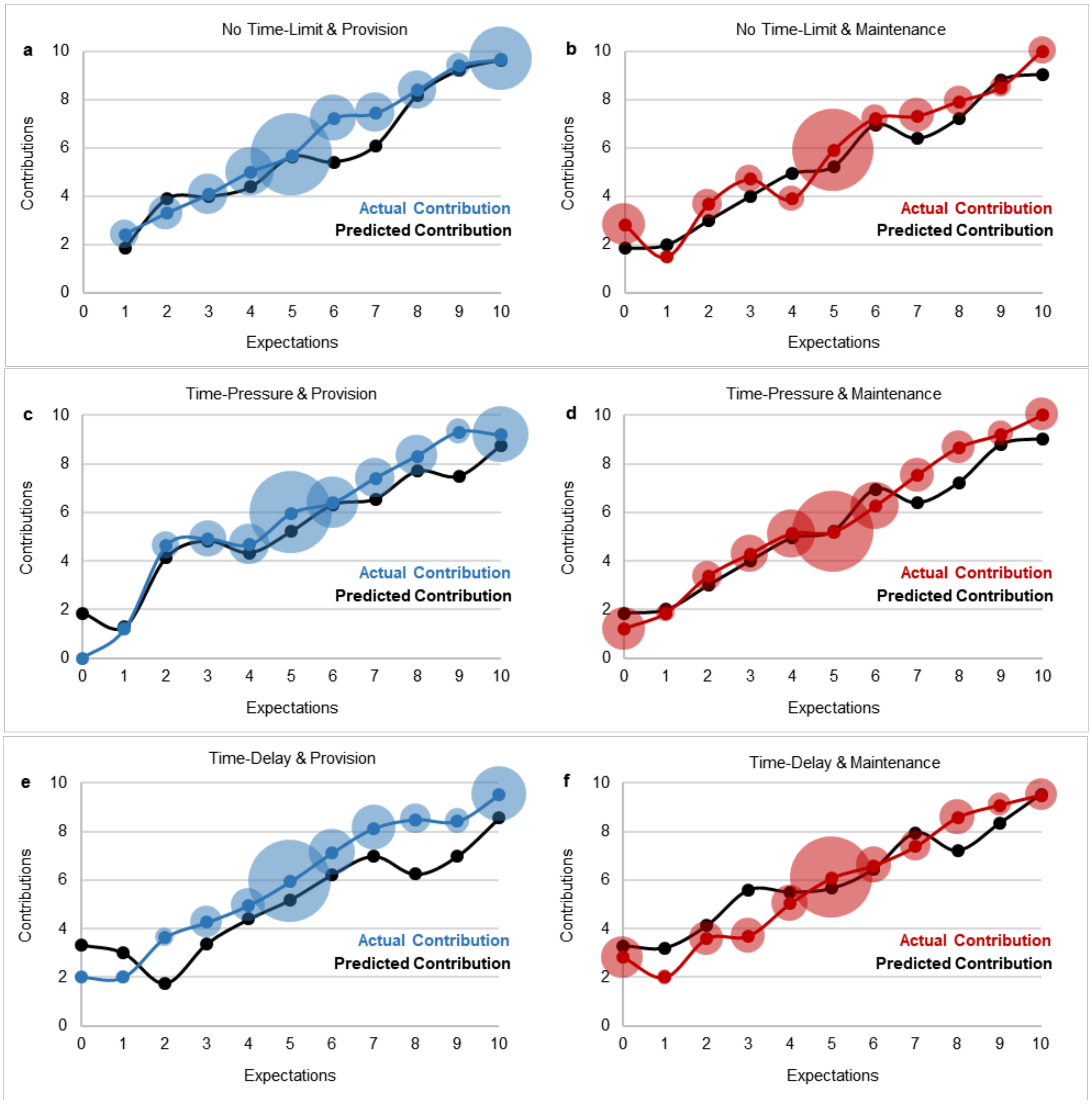

Fig. S5. Actual and predicted cooperation by expectations among participants without correct social dilemma understanding. a, b, Study 1 only; **c – f,** Study 1 and 2 pooled. Included are only participants who incorrectly answered either of the two understanding questions (i.e., the correct strategy for maximizing own monetary gain and for maximizing group monetary gain). **a**, Participants under no time-limit (NTL) in provision dilemma (P). **b**, Participants under NTL in maintenance dilemma (M). **c**, Participants under time-pressure (TP) in P. **d**, Participants under TP in M. **e**, Participants under time-delay (TD) in P. **f**, Participants under TD in M. Colored lines show average actual contribution at each expectation level reported by participants. The size of the bubble indicates the number of participants with respective expectation level. The black lines denote average predicted contribution found by combining expectation with the deliberated contribution preference schedule (Methods).

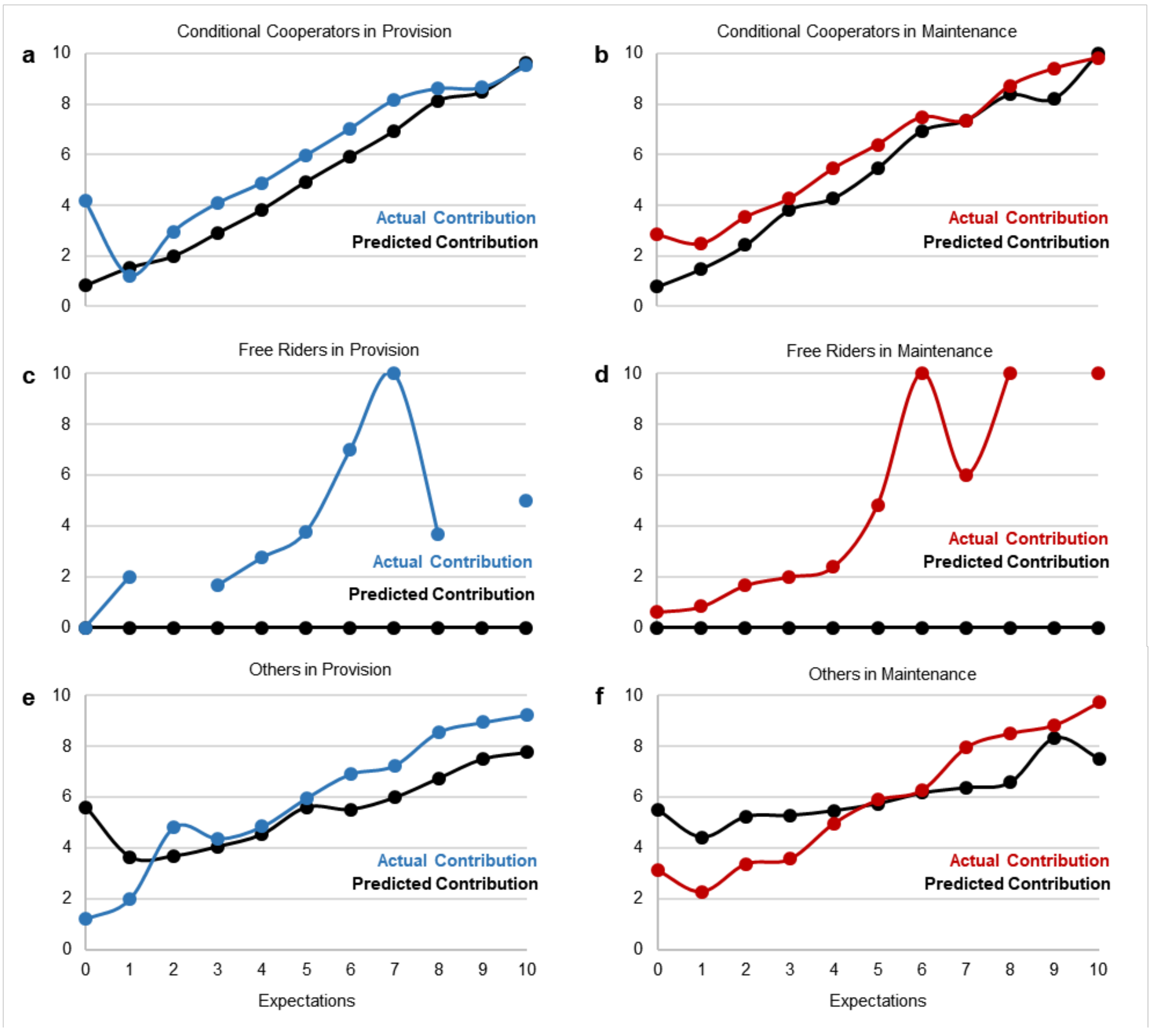

Fig. S6. Actual and predicted contributions by expectations among conditional cooperators, free riders and others (Study 1 & 2 pooled). Colored lines show average actual contribution at each expectation level reported by participants. The black lines denote average predicted contribution found by combining expectation with the deliberated contribution preference schedule (Methods). **a**, Conditional cooperators in provision dilemma (P). **b**, Conditional cooperators in maintenance dilemma (M). **c**, Free riders in P. **d**, Free riders in M. **e**, Others in P. **f**, Others in M.

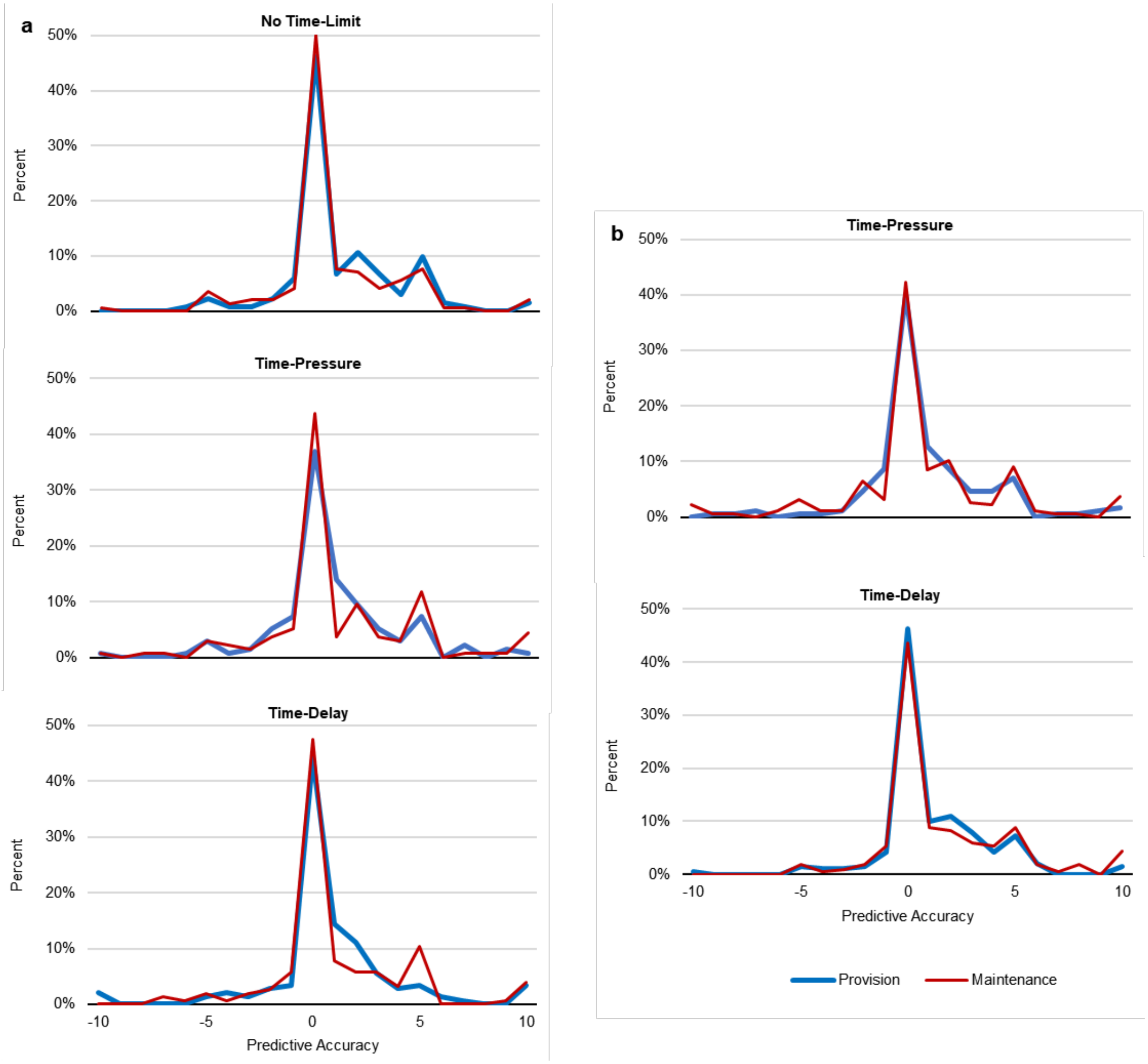

Fig. S7. Accuracy of strong reciprocity measures in predicting cooperation among participants with correct social dilemma understanding. Included are only participants who correctly answered the two understanding questions (i.e., the correct strategy for maximizing own monetary gain and for maximizing group monetary gain). **a**, Study 1: Distribution of predictive accuracy (the difference between actual and predicted contributions for each participant) in NTL, TP and TD. **b**, Study 2: Distribution of predictive accuracy in TP and TD. Note that zero indicates highest predictive accuracy possible.

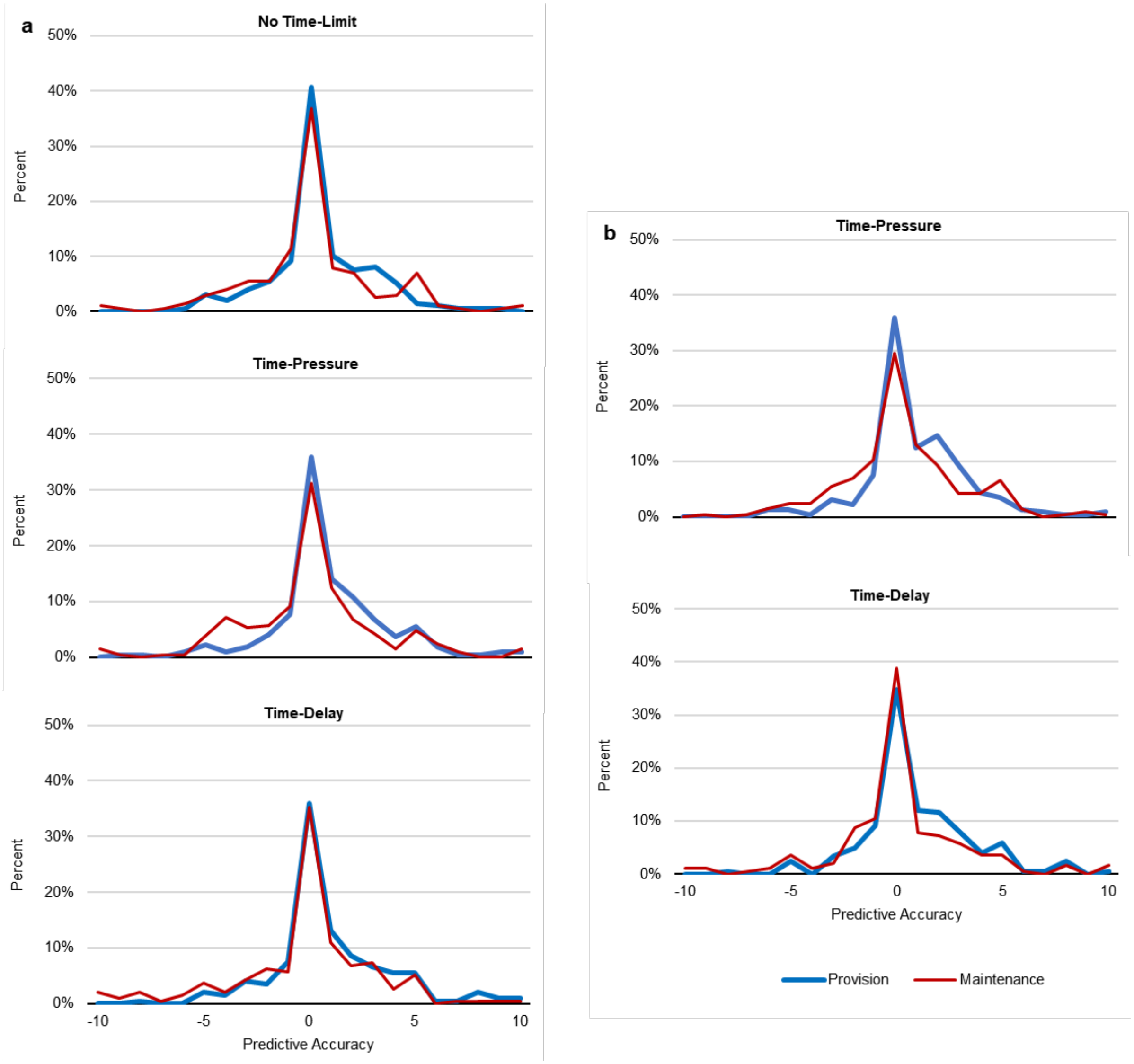

Fig. S8. Accuracy of strong reciprocity measures in predicting cooperation among participants without correct social dilemma understanding. Included are only participants who incorrectly answered either of the two understanding questions (i.e., the correct strategy for maximizing own monetary gain and for maximizing group monetary gain). **a**, Study 1: Distribution of predictive accuracy (the difference between actual and predicted contributions for each participant) in NTL, TP and TD. **b**, Study 2: Distribution of predictive accuracy in TP and TD. Note that zero indicates highest predictive accuracy possible.

2. Supplementary Tables

Table S1. Linear estimates of the relationship between contributions and response time by time limit condition (Study 1).

	**NTL**	**TP**	**TD**
	(1)	(2)	(3)
Dilemma (0 if P, 1 if M)	-0.541**	-0.758***	-0.633***
	(0.231)	(0.224)	(0.221)
$\log_{10}$(RT)	-1.968**	-2.420***	0.692
	(0.826)	(0.731)	(0.478)
Dilemma*$\log_{10}$(RT)	3.166***	3.452***	1.196
	(1.044)	(1.107)	(0.785)
Constant	6.599***	6.675***	6.793***
	(0.157)	(0.147)	(0.142)
	$F(3, 668) = 4.72$	$F(3, 694) = 8.53$	$F(3, 682) = 7.35$
Prob > F	.003	< .001	< .001

Corresponding to Figure 2a, OLS models of public good contributions on dilemma type and median-centered log of RT [log(RT)-log(*MD*)] estimated for each time-limit condition in tokens: no time-limit (NTL), time-pressure (TP) and time-delay (TD). Median RT is 6.6s in NTL, 5.2s in TP and 24.3s in TD. Robust SE in parentheses. * $P < .10$, ** $P < .05$, *** $P < .01$. See Supplementary Information 1.3 for further details.

Table S2. Linear estimates of the relationship between contributions and response time by time limit condition (Study 2).

	**TP**<br>(1)	**TD**<br>(2)
Dilemma (0 if P, 1 if M)	-0.769***<br>(0.206)	-0.083***<br>(0.213)
$\log_{10}(RT)$	-1.406**<br>(0.653)	-0.560<br>(0.501)
Dilemma* $\log_{10}(RT)$	3.232***<br>(1.033)	1.728***<br>(0.799)
Constant	6.552***<br>(0.129)	6.539***<br>(0.142)
	F(3, 795) = 7.74	F(3, 794) = 1.82
Prob > F	< .001	0.142

Corresponding to Figure 2b, OLS models of public good contributions on dilemma type and median-centered log of RT [log(RT)-log(MD)] estimated for time-pressure (TP) and time-delay (TD) conditions in tokens. Median RT is 4.5s in TP and 25.9s in TD. Robust SE in parentheses. * $P < .10$, ** $P < .05$, *** $P < .01$. See Supplementary Information 1.3 for further details.

Table S3. Linear models of actual contributions on predicted contributions, expectations, understanding, experience, CRT score and demographic variables by experimental conditions.

	Provision					Maintenance				
	Study 1			Study 2		Study 1			Study 2	
	NTL	TP	TD	TP	TD	NTL	TP	TD	TP	TD
	(1)	(2)	(3)	(4)	(5)	(6)	(7)	(8)	(9)	(10)
Predicted contribution	0.322***	0.231***	0.126**	0.225***	0.340***	0.295***	0.063	0.249***	0.122**	0.224***
	(0.075)	(0.058)	(0.054)	(0.057)	(0.058)	(0.057)	(0.045)	(0.061)	(0.052)	(0.057)
Expectation	0.543***	0.602***	0.690***	0.509***	0.513***	0.510***	0.808***	0.495***	0.721***	0.623***
	(0.087)	(0.074)	(0.064)	(0.070)	(0.071)	(0.071)	(0.049)	(0.072)	(0.057)	(0.066)
Understanding	0.480**	0.117	-0.087	-0.286	-0.136	0.609**	0.778***	0.555**	0.410	0.735***
	(0.219)	(0.241)	(0.228)	(0.215)	(0.206)	(0.257)	(0.261)	(0.271)	(0.255)	(0.246)
Experience	0.491	0.209	0.060	0.146	-0.484	0.153	0.603*	1.012**	-0.080	0.055
	(0.340)	(0.386)	(0.469)	(0.365)	(0.313)	(0.371)	(0.355)	(0.496)	(0.312)	(0.378)
CRT score				0.171*	0.138				0.202*	-0.094
				(0.090)	(0.084)				(0.113)	(0.113)
Gender (1 if female)	0.355	-0.341	-0.467*	-0.430*	0.435**	-0.195	-0.002	0.025	0.531**	0.134
	(0.258)	(0.259)	(0.272)	(0.231)	(0.212)	(0.285)	(0.288)	(0.345)	(0.259)	(0.245)
Age	-0.020**	-0.005	-0.017*	0.006	0.023***	-0.007	-0.021**	-0.009	-0.007	-0.003
	(0.009)	(0.010)	(0.010)	(0.008)	(0.009)	(0.012)	(0.009)	(0.012)	(0.009)	(0.010)
Constant	1.750***	2.042***	2.931***	2.225***	0.429	2.053***	1.890***	2.140***	1.050***	1.740***
	(0.507)	(0.463)	(0.535)	(0.440)	(0.4460)	(0.650)	(0.529)	(0.634)	(0.457)	(0.545)
F	F(6,322)=109.70	F(6,348)=57.87	F(6,335)=48.32	F(7,390)=41.37	F(7,391)=93.10	F(6,336)=38.27	F(6,336)=78.83	F(6,337)=34.53	F(7,393)=69.73	F(7,391)=53.50
Prob > F	< .001	< .001	< .001	< .001	< .001	< .001	< .001	< .001	< .001	< .001
Adjusted R^2	0.557	0.452	0.453	0.428	0.521	0.438	0.522	0.363	0.486	0.461

OLS models of public good contributions on predicted contributions, expected contributions, social dilemma understanding (i.e., correct answers on self-gain and group-gain maximization questions), experience (i.e., previous participation in lab studies or prior knowledge of the public good game), CRT scores (ranging from 0 to 3; higher scores indicating tendency for deliberated thinking; elicited only in Study 2) and demographic variables (i.e., gender and age), estimated in tokens for each social dilemma (provision and maintenance) and time-limit condition: no time-limit (NTL), time-pressure (TP) and time-delay (TD). Robust SE in parentheses. * $P < .10$, ** $P < .05$, *** $P <$ 01.

Table S4. Models of strong reciprocity on experimental conditions, understanding, social appropriateness, CRT score, experience and demographic variables.

	Expectations		Preferences					
			Conditional Cooperators		Free Riders		Others	
	Study 1 β (1)	Study 2 β (2)	Study 1 OR (3)	Study 2 OR (4)	Study 1 OR (5)	Study 2 OR (6)	Study 1 OR (7)	Study 2 OR (8)
Dilemma (0 if P, 1 if M)	-0.485***	-0.510***	0.726***	0.712***	1.927**	1.658	1.290**	1.357***
	(0.107)	(0.126)	(0.071)	(0.079)	(0.587)	(0.524)	(0.128)	(0.156)
Time-Limit (0 if TP)								
NTL	-0.334***		0.842		1.068		1.183	
	(0.123)		(0.095)		(0.324)		(0.134)	
TD	-0.143	0.018	0.944	0.946	0.773	1.107	1.093	1.043
	(0.121)	(0.120)	(0.106)	(0.100)	(0.252)	(0.327)	(0.123)	(0.114)
Self-gain understanding	-0.942***	-0.912***	1.194*	1.341**	4.424***	3.996***	0.712***	0.646***
	(0.113)	(0.132)	(0.123)	(0.156)	(1.686)	(1.774)	(0.074)	(0.077)
Group-gain understanding	1.223***	1.475***	1.620***	1.721***	1.249	1.101	0.601***	0.572***
	(0.121)	(0.141)	(0.179)	(0.217)	(0.416)	(0.477)	(0.067)	(0.073)
Appropriateness: free-riding	-0.594***		0.747***		1.732**		1.213*	
	(0.126)		(0.077)		(0.427)		(0.125)	
Appropriateness: equal split	-1.383***		0.929		2.069*		0.994	
	(0.125)		(0.107)		(0.863)		(0.116)	
Appropriateness: complete cooperatio	0.409***		1.359***		0.841		0.754***	
	(0.112)		(0.146)		(0.235)		(0.08)	
CRT score		-0.017		1.165***		1.502***		0.809***
		(0.053)		(0.056)		(0.180)		(0.040)
Experience	0.094	-0.052	1.437**	0.891	1.220	1.386	0.672***	1.045
	(0.167)	(0.206)	(0.217)	(0.152)	(0.476)	(0.524)	(0.103)	(0.184)
Gender (1 if female)	0.118	-0.013	0.865	1.114	0.705	0.440***	1.217*	1.012
	(0.121)	(0.126)	(0.092)	(0.126)	(0.197)	(0.132)	(0.131)	(0.119)
Age	0.003	0.009*	0.989***	0.993	1.022**	0.999	1.008*	1.008*
	(0.005)	(0.005)	(0.004)	(0.004)	(0.011)	(0.012)	(0.004)	(0.005)
Constant	5.226***	4.977***	1.119***	1.132	0.004***	0.006***	0.907	0.842
	(0.247)	(0.242)	(0.248)	(0.252)	(0.003)	(0.005)	(0.203)	(0.191)
F	F(11,2056)=50.94	F(8,1588)=24.49	χ^2(11,2056)=121.53	χ^2(8,1597)=77.10	χ^2(11,2056)=61.64	χ^2(8,1597)=37.17	χ^2(11,2056)=125.35	χ^2(8,1597)=102.24
Prob > F	< .001	< .001	< .001	< .001	< .001	< .001	< .001	< .001
Adjusted or pseudo R^2	0.203	0.096	0.048	0.038	0.103	0.109	0.047	0.052

OLS models of expected contributions (Models 1 and 2) and logit models of likelihood of being categorized as conditional cooperator (Models 3 and 4), free rider (Models 5 and 6) or other (Models 7 and 8) on experimental conditions, cognition measures including social dilemma understanding (i.e., correct answers on self-gain and group-gain maximization questions), social appropriateness ratings (ranging from -1 to 1; higher scores indicating higher perceived appropriateness; elicited only in Study 1) and CRT scores (ranging from 0 to 3; higher scores indicating tendency for deliberated thinking; elicited only in Study 2), as well as experience (i.e., previous participation in lab studies or prior knowledge of the public good game) and demographic variables (i.e., gender and age), estimated for Study 1 and 2 separately. OLS models report regression coefficients (β) and adjusted R^2, logit models report odds ratios (OR) and pseudo R^2. Robust SE in parentheses. * $P < .10$, ** $P < .05$, *** $P < 01$.

3. Supplementary Results

3.1. Detailed Results

Study 1

Contributions

No Time-Limit Conditions. Average tokens contributed to the public good (H_1) was significantly higher in P (M = 6.58, SD = 2.92, 95% CI = [6.26, 6.89]) than in M (M = 6.12, SD = 3.05, 95% CI = [5.80, 6.44]), $t(670) = 1.99$, $P = .048$, $d = 0.153$.

Time-Limit Conditions. ANOVA on contributions showed: (H_1) significant difference between P (M = 6.73, SD = 2.73, 95% CI = [6.52, 6.93]) and M (M = 6.01, SD = 3.14, 95% CI = [5.78, 6.25]), $F(1, 1380) = 20.34$, $P < .001$, $\eta_p^2 = .015$; (H_2) no difference between TP (M = 6.31, SD = 3.00, 95% CI = [6.09, 6.53]) and TD (M = 6.43, SD = 2.93, 95% CI = [6.22, 6.65]), $F(1, 1380) = 0.68$, $P = .408$, $\eta_p^2 = .000$; and (H_3) no significant interaction, $F(1, 1380) = 0.05$, $P = .825$, $\eta_p^2 < .001$.

Conditional Cooperation

No Time-Limit Conditions. The proportion of conditional cooperators (H_4) in the NT sample was significantly higher in P (57.4%) compared to M (46.9%), χ^2 (1, n = 672) = 7.43, $P = .006$.

Time-Limit Conditions. χ^2 tests indicated: significantly higher prevalence of conditional cooperators (H_4) in P (60.7%) than in M (49.5%), χ^2 (1, n = 1384) = 17.54, $P < .001$; and (H_7) no difference between TP (55.4%) and TD (54.8%), χ^2 (1, n = 1384) = 0.06, $P = .813$. The Logit model on prevalence of conditional cooperators, χ^2 (3, n = 1384) = 17.61, $P < .001$, indicated no interaction (H_{10}), OR = 1.08, 95% CI = [0.71, 1.66], $P = .717$. As elicitation of preference for conditional cooperation was not time-limited, H_7 and H_{10} are tests of spill-over effects due to previous time-limits.

Expectations

No Time-Limit Conditions. In the overall no time-limit sample, expectations about group members' contributions to the public good (H_5) was significantly higher in P (M = 5.78, SD = 2.53, 95% CI = [5.50, 6.05]) than in M (M = 4.93, SD = 2.63, 95% CI = [4.65, 5.21]), $t(670) = 4.25$, $P < .001$, $d = 0.328$. Among conditional cooperators, expectations were also

significantly higher in P (*M* = 5.64, *SD* = 2.56, 95% CI = [5.27, 6.01]) than in M (*M* = 4.99, *SD* = 2.74, 95% CI = [4.56, 5.41]), *t*(348) = 2.30, *P* = .022, *d* = 0.247.

Time-Limit Conditions. ANOVA on expectations in the overall time-limit sample found: (H_5) a significant difference between P (*M* = 6.01, *SD* = 2.28, 95% CI = [5.84, 6.18]) and M (*M* = 5.08, *SD* = 2.64, 95% CI = [4.89, 5.28]), F(1, 1380) = 48.30, *P* < .001, η_p^2 = .034; (H_8) no difference between TP (*M* = 5.61, *SD* = 2.52, 95% CI = [5.42, 5.79]) and TD (*M* = 5.49, *SD* = 2.50, 95% CI = [5.31, 5.68]), F(1, 1380) = 0.61, *P* = .435, η_p^2 < .001; and (H_{11}) no interaction, F(1, 1380) = 0.15, *P* = .701, η_p^2 < .001. Similarly, expectations of conditional cooperators (H_5) were significantly higher in P (*M* = 6.26, *SD* = 2.23, 95% CI = [6.05, 6.48]) than in M (*M* = 5.22, *SD* = 2.63, 95% CI = [4.94, 5.50]), F(1, 759) = 34.72, *P* < .001, η_p^2 = .044; while there was (H_8) no difference between TP (*M* = 5.87, *SD* = 2.47, 95% CI = [5.63, 6.12]) and TD (*M* = 5.72, *SD* = 2.47, 95% CI = [5.47, 5.97]), F(1, 759) = 0.46, *P* = .499, η_p^2 < .001; and (H_{11}) no interaction, F(1, 759) = 0.58, *P* = .446, η_p^2 < .001.

Predicted Contributions

No Time-Limit Conditions. Predicted contributions (H_6) did not significantly differ between P (*M* = 5.92, *SD* = 2.96, 95% CI = [5.60, 6.25]) and M (*M* = 5.76, *SD* = 3.13, 95% CI = [5.43, 6.10]), *t*(670) = 0.68, *P* = .496, *d* = 0.053.

Time-Limit Conditions. ANOVA on predicted contributions shows: (H_6) no difference between P (*M* = 5.87, *SD* = 2.91, 95% CI = [5.65, 6.08]) and M (*M* = 5.63, *SD* = 3.14, 95% CI = [5.39, 5.86]), F(1, 1380) = 2.14, *P* = .144, η_p^2 = .002; (H_9) no difference between TP (*M* = 5.69, *SD* = 3.07, 95% CI = [5.47, 5.92]) and TD (*M* = 5.80, *SD* = 2.98, 95% CI = [5.58, 6.03]), F(1, 1380) = 0.47, *P* = .494, η_p^2 < .001; and (H_{12}) no significant interaction, F(1, 1380) = 0.20, *P* = .653, η_p^2 < .001.

Study 2

Contributions

ANOVA on *contributions* showed: (H_1) significant difference between P (*M* = 6.56, *SD* = 2.68, 95% CI = [6.38, 6.75]) and M (*M* = 6.14, *SD* = 3.18, 95% CI = [5.91, 6.36]), F(1, 1593) = 8.41, *P* = .004, η_p^2 = .005; (H_2) significant difference between TP (*M* = 6.20, *SD* = 2.93, 95% CI = [6.00, 6.40]) and TD (*M* = 6.50, *SD* = 2.97, 95% CI = [6.29, 6.70]), F(1, 1593) = 4.09, *P* = .043, η_p^2 = .003; and (H_3) a significant interaction, F(1, 1593) = 3.99, *P* = .046, η_p^2 = .002.

According to post hoc tests, average contributions in the M dilemma were significantly lower under TP (M = 5.84, SD = 3.20, 95% CI = [5.53, 6.15]) than under TD (M = 6.43, SD = 3.14, 95% CI = [6.12, 6.74]), $t(798) = 2.63$, $P < .001$, $d = 0.186$. In the P dilemma, time-limits had no effect on contributions (TP: M = 6.56, SD = 2.57, 95% CI = [6.31, 6.81]; TD: M = 6.56, SD = 2.78, 95% CI = [6.29, 6.84]), $t(795) = 0.02$, $P = .985$, $d = 0.001$. From the alternative perspective, contributions under TP were significantly lower in the M dilemma than in the P dilemma, $t(797) = 3.50$, $P < .001$, $d = 0.248$. Under TD, dilemma type had no effect on contributions, $t(796) = 0.63$, $P = .527$, $d = 0.045$.

Conditional Cooperation

χ^2 tests indicated: (H_4) a significantly higher prevalence of conditional cooperators in P (66.2%) than in M (57.3%), χ^2 (1, n = 1597) = 13.69, $P < .001$; and (H_7) no difference between TP (61.7%) and TD (61.8%), χ^2 (1, n = 1597) = 0.00, $P = .975$. The Logit model on prevalence of conditional cooperators, χ^2 (3, n = 1597) = 14.49, $P = .002$, indicated no interaction (H_{10}), OR = 0.82, 95% CI = [0.55, 1.23], $P = .346$. Note that the elicitation of preferences for conditional cooperation was not time limited.

Expectations

ANOVA on expectations in the overall Study 2 sample found: (H_5) a significant difference between P (M = 6.00, SD = 2.28, 95% CI = [5.84, 6.16]) and M (M = 5.08, SD = 2.56, 95% CI = [4.89, 5.26]), $F(1, 1593) = 55.16$, $P < .001$, $\eta_p^2 = .033$; (H_8) no difference between TP (M = 5.50, SD = 2.56, 95% CI = [5.32, 5.67]) and TD (M = 5.58, SD = 2.47, 95% CI = [5.41, 5.75]), $F(1, 1593) = 0.43$, $P = .510$, $\eta_p^2 < .001$; and (H_{11}) no interaction, $F(1, 1593) = 0.21$, $P = .648$, $\eta_p^2 < .001$. Likewise, expectations of conditional cooperators (H_5) were significantly higher in P (M = 5.99, SD = 2.26, 95% CI = [5.80, 6.21]) than in M (M = 5.28, SD = 2.67, 95% CI = [5.06, 5.58]), $F(1, 982) = 20.36$, $P < .001$, $\eta_p^2 = .021$; while (H_8) there was no difference between TP (M = 5.71, SD = 2.53, 95% CI = [5.49, 5.94]) and TD (M = 5.60, SD = 2.44, 95% CI = [5.39, 5.82]), $F(1, 982) = 0.63$, $P = .427$, $\eta_p^2 < .001$; and (H_{11}) no interaction, $F(1, 982) = 0.01$, $P = .925$, $\eta_p^2 < .001$.

Predicted Contributions

ANOVA on predicted contributions showed: (H_6) no difference between P (M = 5.60, SD = 2.97, 95% CI = [5.39, 5.81]) and M (M = 5.42, SD = 3.14, 95% CI = [5.20, 5.64]), $F(1, 1593) = 1.36$, $P = .244$, $\eta_p^2 < .001$; (H_9) no difference between TP (M = 5.46, SD = 3.01,

95% CI = [5.26, 5.67]) and TD (*M* = 5.56, *SD* = 3.11, 95% CI = [5.34, 5.77]), F(1, 1593) = 0.36, *P* = .550, $\eta_p^2 < .001$; and (H_{12}) no significant interaction, F(1, 1593) = 0.70, *P* = .403, $\eta_p^2 < .001$.

Social Appropriateness

Comparing M and P, freeriding is found to be significantly less socially appropriate when it involves contributing 0 tokens to the project (i.e., P dilemma: *M* = -0.733, *SD* = 0.390, 95% CI = [-0.76, -0.71]) than when it involve withdrawing 10 tokens from the project (i.e., M dilemma: *M* = -0.496, *SD* = 0.562, 95% CI = [-0.53, -0.46]), *t*(2054) = -11.09, *p* < 0.0001, *d* = -0.489.

In contrast, equal-split of the endowment is significantly more socially appropriate when it involves contributing 5 tokens to the project (i.e., P dilemma: *M* = 0.386, *SD* = 0.387, 95% CI = [0.36, 0.41]) than when it involves withdrawing 5 tokens from the project (i.e., M dilemma: *M* = 0.318, *SD* = 0.44, 95% CI = [0.29, 0.34]), *t*(2054) = 3.77, *p* = 0.0002, *d* =0.166.

Likewise, full cooperation in the PGG is more socially appropriate when it involves contributing all of the endowment to the project (i.e., P dilemma: *M* = 0.724, *SD* = 0.413, 95% CI = [0.70, 0.75]) than when it involves keeping all of the endowment in the project (i.e., M dilemma: *M* = 0.622, *SD* = 0.543, 95% CI = [0.59, 0.66]), *t*(2054) = 4.78, *p* < 0.0001, *d* = 0.211.

Cognitive Reflection Test

The standard Cognitive Reflection Test (1) (CRT), consisting of three questions (Q1, Q2 and Q3), was administered at the end of Study 2 (see Experimental Instructions below for the questions). We calculated two types of CRT scores for each individual. The standard CRT scores (ranging from 0 to 3), analyzed in the main text and used below, indicated the total number of correct answers (i.e., 5 for Q1, 5 for Q2 and 47 for Q3). The alternative iCRT (2) scores (also between 0 to 3), analyzed in the main text, indicated the number of intuitive but incorrect answers (i.e., 10 for Q1, 100 for Q2 and 24 for Q3).

The distribution of the CRT scores was in line with previous findings (3) with 36.3% of Study 2 participants scoring 0, 19.0% scoring 1, 20.7% scoring 2 and 24.1% scoring 3. 37.1% of participants reported previously seeing at least one of the three CRT questions. Although experience has previously been shown not to improve performance on the CRT (4), our results indicate the opposite, with significantly higher scores among those with self-reported experience or knowledge (*M* = 1.80, *SD* = 1.17, 95% CI = [1.70, 1.89]) than among others (*M* = 1.04, *SD* = 1.12, 95% CI = [0.98, 1.11]), *t*(1595) = 12.79, *P* < .001, *d* = 0.663.

We detected significant differences in CRT scores between P and M. Specifically, a two-way ANOVA indicated CRT scores were significantly higher in P (*M* = 1.41, *SD* = 1.23, 95% CI = [1.32, 1.49]) than in M (*M* = 1.24, *SD* = 1.16, 95% CI = [1.16, 1.32]), F(1, 1593) = 7.32, *P* = .007, but there was neither a significant difference between TP (*M* = 1.28, *SD* = 1.19, 95% CI = [1.19, 1.36]) and TD (*M* = 1.37, *SD* = 1.20, 95% CI = [1.29, 1.46]), F(1, 1593) = 2.69, *P* = .101, nor an interaction between time-limits and dilemma types, F(1, 1593) = 0.98, *P* = .321. In addition, CRT scores showed a small but positive correlation with cooperation (r_s = .07, P = .008).

In two supplementary studies, we checked whether the difference in CRT scores between P and M was due to sampling error. First, we ran simulations that randomly assigned participants' initial CRT scores to one of two hypothetical treatments—mimicking random assignment to social dilemma types—and then tested for differences in average scores between the hypothetical treatments, a procedure that was repeated 3000 times. The difference in CRT scores was significant in less than 5% of the tests, indicating acceptably low probability of sampling bias. Second, as described in the main text, we reinvited 50% of Study 2 participants one and a half years later only to complete the CRT a second time. While the initial CRT scores among the re-invited participants were 9.7% higher for those in P than in M during Study 2, $\chi^2(3) = 9.95$, *P* = .019, no difference was detected in their second CRT scores, χ^2 (3) = 2.71, *P* = .439. Consistent with previous findings (5), the second CRT scores (*M* = 1.53, *SD* = 1.22, 95% CI = [1.44, 1.61]) were somewhat higher than the initial CRT scores (*M* = 1.35, *SD* = 1.20, 95% CI = [1.26, 1.43]), indicating possible learning over time. Nevertheless, the mean score of 1.53 achieved over time was far from the maximum score of 3, suggesting that our results were not driven by a ceiling effect. Hence, by ruling out sampling bias, these supplementary studies indicated that exposure to social dilemma type had a spill-over effect on CRT scores.

We preregistered two hypotheses (H_{13} and H_{14}) about the CRT scores (Methods). However, the significant spill-over of dilemma conditions on CRT scores invalidated the tests of these hypotheses. Despite their invalidity, we report the analyses intended to test these hypotheses for completeness, but they should be interpreted with caution.

We hypothesized (H_{13}) that contributions in P would be higher than M among participants with a strong tendency to decide intuitively. Average contributions in P (*M* = 6.31, *SD* = 2.46, 95% CI = [6.02, 6.60]) was higher than M (*M* = 5.96, *SD* = 2.98, 95% CI = [5.62,

6.30]) among those with initial CRT scores of zero, but this difference failed to reach statistical significance, $t(578) = 1.53$, $P = .127$, $d = 0.127$. When considering those who scored zero the second time the CRT was elicited, contributions were again higher in P ($M = 6.26$, $SD = 2.43$, 95% CI = [5.84, 6.67]) than in M ($M = 5.68$, $SD = 2.82$, 95% CI = [5.16, 6.20]), and the difference tended towards statistical significance, $t(246) = 1.73$, $P = .085$, $d = 0.221$.

Finally, we hypothesized (H_{14}) that the difference in contributions between P and M would decrease as CRT scores increase. Using OLS regressions of contributions on dilemma type and CRT scores, we failed to find evidence for the hypothesis—operationalized as the test of interaction between dilemma type and CRT score—either using the initial ($P = .512$) or the second CRT scores ($P = .454$).

3.2. Tests of Decision Conflict Accounts

We evaluate here the evidence in our data for and against the decision conflict accounts of response times (RTs). Some of the exploratory support we found for SHH and SCA depends upon fast RTs of cooperative decisions being interpreted as indicating intuition. Recently it has been argued that RTs in social dilemmas may instead reflect decision conflict—increasing with the subjective difficulty of making a choice (6, 7). These accounts predict that (i) TP will increase random decision error (8-10), that (ii) TP will increase congruency of choices with preferences (11), and that (iii) longer RTs will be associated with fewer extreme decisions (12). Our data does not provide clear evidence for any of these predictions, as detailed below.

(i) Single-process accounts such as the drift diffusion model (DDM) (8) formalizes decision-making as a continuous process of evidence accumulation. Therefore, DDM can and has been interpreted as modeling the process of deliberation. These models imply or assume that artificially cutting evidence accumulation short, for example via time-pressure, should increase random decision error (8, 10). In the public goods game framework, DDM therefore predicts contributions in TP to converge towards the 50% contribution level as compared to TD. Despite some evidence in support of this view (9), other studies that found intuitive cooperation well above the 50% level contradict this prediction (11, 13). Similar to these other studies, which rely on Provision dilemmas, we did not find support for the decision error account in the P dilemma: the difference in contributions between TP and TD conditions in P was only 0.5%, $t(1492) = 0.32$, $P = .748$. On the other hand, average contributions in M were closer to the 50% level in TP ($M = 5.88$) than in TD ($M = 6.28$), $t(1485) = 2.41$, $P = .016$, $d = 0.125$. Nevertheless, the lack of evidence for the decision error account in our P samples

(as well as in the wider literature) and the positive effect of understanding on public good game contributions in our M samples (Supplementary Table 3) suggests that the negative effect of TP on cooperation observed in M is likely not due to increased error.

(ii) In light of the above-mentioned evidence against DDM, Chen and Krajbich (11) revised the standard DDM by assuming that evidence accumulation will be biased towards one's preferences at the outset of the decision-making process. Their Biased Drift Diffusion Model (BDDM) predicts that time-pressure will promote congruence of decisions with preference for cooperation or defection. Supporting this hypothesis, Chen and Krajbich (11) found that time-pressure increases congruence both for prosocial and selfish participants in modified dictator games, whereas Mischkowski and Glöckner (14) found a similar effect only among prosocial subjects. To test this view, we studied the effect of time-limits on cooperation for each cooperation preference type separately (Supplementary Fig. 3). Two-way ANOVA models of cooperation, which include dilemma type, time-limits and their interactions as factors, and which found confirmatory evidence for SCA in Study 2, were insignificant for conditional cooperators, $F(3, 982) = 0.96$, $P = .410$ as well as for free-riders, $F(3, 44) = 1.33$, $P = .278$. Instead, the effect of time-limits was solely due to those classified as "others", whose contributions were significantly higher in P ($M = 6.61$) than in M ($M = 5.94$), $F(1, 559) = 8.48$, $P = .004$, $\eta_p^2 = .015$, and significantly higher TD ($M = 6.61$) than in TP ($M = 5.87$), $F(1, 559) = 9.90$, $P = .002$, $\eta_p^2 = .017$. Hence, we find no evidence for BDDM in our data.

(iii) Consistent with BDDM, Evans, Dillon and Rand (12) also predict that extreme decisions (i.e., full cooperation or free riding) would be more prevalent than intermediate decisions among fast than among slow responders. To compare extreme and intermediate decisions, we create a new binary variable, EXT, which takes on value 1 for 0 or 10 tokens contributed to the public good game and takes value 0 otherwise. We used the pooled data and we estimated a multiple Logit model of EXT on the binary dilemma condition variable, $\log_{10}(RT)$ and their interaction. The EXT model was statistically significant overall (χ^2 (3, n = 3653) = 13.49, $P = .004$), with a significant interaction term ($OR = 1.60$, 95% CI = [1.12, 2.29], $P = .011$). Contrary to the prediction of fast extreme decisions, the prevalence of extreme decisions did not change over time in the P dilemma ($OR = 0.90$, 95% CI = [0.69, 1.16], $P = .405$). Moreover, extreme decisions became significantly more likely over time in the M dilemma ($OR = 1.43$, 95% CI = [1.11, 1.84], $P = .005$). In short, our tests do not provide consistent evidence for any of the predictions based on the decision conflict accounts.

Chen and Krajbich's BDDM (11) makes the simplifying assumption that priors reflect one's preferences—an assumption that arguably keeps their model within the single-process framework. However, in our context where intuitions (selfish vs. prosocial) are distinct from preferences (e.g., for strong reciprocity), priors can instead be thought of as corresponding to intuitions. This *revised* version of BDDM (i.e., R-BDDM) is consistent in its predictions with our generalized dual-process account of cooperation (ISR; see main text), predicting a decrease in contributions with RT when intuitions are relatively more selfish (as in the M dilemma) and an increase in contributions with RT when heuristics are relatively more prosocial (as in the P dilemma). R-BDDM can be estimated for our overall sample using linear interaction models of public good game contributions (C) on the binary dilemma condition variable, $\log_{10}$(RT) and their interaction (see Supplementary Tables 1 and 2), which correspond to the exploratory results supporting ISR that are presented in the main text and further detailed here in the next section.

3.3. Description of the Linear Models of Response Times

We model the relationships between RT and cooperation using linear regressions of tokens contributed to the public good on dilemma type, median-centered $\log_{10}$(RT) and their interaction. We estimate a model for each study and each time-limit condition separately.

Study 1. Consistent with the time-trends seen in Fig. 2 and as summarized on Supplementary Table 1, contributions in the P dilemma showed a significant decrease over time for NTL (b = -1.97, P = 0.017) and TP (b = -2.42, P = 0.001) conditions but not for TD (b = 0.69, P = 0.148), whereas contributions in the M dilemma tended to increase over time, which was significant for TD (NTL: b = 1.20, P = 0.061; TP: b = 1.03, P = 0.215; TD: b = 1.89, P = 0.003). The interaction terms indicated contrasting trends between M and P in the NTL (b = 3.17, P = 0.003) and TP (b = 3.45, P = 0.002) conditions, but not in the TD condition (b = 1.20, P = 0.128).

Study 2. In line with the time-trends observed on Fig. 2 and as summarized on Supplementary Table 2, cooperation in TP tended to weaken over time in the P dilemma (b = -1.41, P = 0.032), and tended to grow stronger over time in the M dilemma (b = 1.83, P = 0.023). The contrasting time-trends between the two dilemmas (tending to be negative in P and positive in M) resulted in interaction (b = 3.23, P = 0.002). Although corresponding but weaker trends could be visually observed in TD, the model for contributions was overall insignificant for the time-delay condition ($F(3, 794) = 1.82$, P = 0.142).

4. Supplementary Methods

Experimental Instructions

- **Slider training (p. 19)**
- **Instructions for the Public Good Game (p. 20)**
- **Part A: Public Good Game Decision Screens (p. 21)**
- **Part B:**
  - **Expectations (p. 24)**
  - **Understanding (p. 28)**
- **Part C: Cooperation Preferences (p. 30)**
- **Part D: Cognitive Reflection Task (p. 33)**

Slider Training

Initial Screen

<u>Slider Practice</u>

In this study, you will use a slider to input your responses. You can practice using the slider on this screen.

The slider below allows you to pick a whole number from 0 to 10.

Please click on the empty bar below, drag your mouse cursor to the value of your choice while holding your click, and release your click to set the value as your response.

After you set the value on the slider you will still need to click "Continue" to finalize the submission of your response.

Now please submit a hypothetical response for practice.

0 10

Follow-up Screen (Displaying Input on Initial Screen)

You submitted the value **5**. Click "Back" for more practice with the slider or click "Continue" to begin reading the instructions of the study.

Instructions for the Public Good Game

Provision

"Please carefully read all of the instructions below before moving on.

You have been randomly assigned to interact with 3 other people. All of you receive this same set of instructions.

Each person in your group is given 10 tokens worth 100 pence in total (1 token = 10 pence).

You must each decide how many of these 10 tokens to contribute to a group project and how many to keep for yourself.

At the end of the interaction, each 10 pence token contributed to the group project will increase the value of the project by 20 pence, which will then be split evenly between the four of you. Thus, for every token contributed to the group project, each group member receives 5 pence.

For example, if everyone keeps 10 tokens for oneself, then the value of the project would be zero and total earnings from the interaction for each person would be 100 pence.

If everyone contributes 10 tokens to the project, then the value of the project would be 800 pence and each of you will earn 200 pence in total.

But if everyone else contributes 10 tokens to the project, while you keep 10 tokens for yourself, you will earn 250 pence, while the others will earn only 150 pence. That is because for every token you contribute to the project, you get only 5 pence back.

Once you and the other people have chosen how many tokens to contribute to the group project, the interaction is over."

Maintenance

"Please carefully read all of the instructions below before moving on.

You have been randomly assigned to interact with 3 other people. All of you receive this same set of instructions.

There are 40 tokens in a group project worth 400 pence in total (1 token = 10 pence).

You must each decide how many of these tokens, up to a maximum of 10, to withdraw for yourself and how many to leave in the group project.

At the end of the interaction, each 10 pence token left in the group project will increase the value of the project by 20 pence, which will then be split evenly between the four of you. Thus, for every token left in the group project, each group member receives 5 pence.

For example, if everyone leaves 10 tokens in the project, then the value of the project would

be 800 pence and total earnings from the interaction for each person would be 200 pence.

If everyone withdraws 10 tokens for themselves, then the value of the project would be zero and each of you will earn 100 pence in total.

But if everyone else leaves 10 tokens in the project, while you withdraw 10 tokens for yourself, you will earn 250 pence, while the others will earn only 150 pence. That is because for every token you leave in the project, you get only 5 pence back.

Once you and the other people have chosen how many tokens to withdraw from the group project, the interaction is over."

Part A: Public Good Game Decision Screens

Provision – No Time-Limit: Preliminary Screen

Part A

Part A will now begin.

Please continue.

Provision – No Time-Limit: Decision Screen

How many tokens do you wish to contribute to the group project?

Your contribution:

0 10

Provision – Time-Pressure: Preliminary screen (displayed for 15s)

Part A

Please continue reading now.

The next screen will appear very soon.

On the next screen, you will need to submit your decision in less than 10 seconds in order to be eligible for any payments.

If you take more than 10 seconds to submit your decision, then you will not get paid.

Provision – Time-Pressure: Decision screen (Note: Study 2 has a 5 second time-limit)

Please be quick. You must submit your decision in less than 10 seconds!

How many tokens do you wish to contribute to the group project?

Your contribution:

0 10

Provision – Time-Delay: Preliminary screen (displayed for 15s)

Part A

Please continue reading now.

The next screen will appear very soon.

On the next screen, you will need to think for more than 10 seconds before submitting your decision in order to be eligible for any payments.

If you submit your decision in less than 10 seconds, then you will not get paid.

Provision – Time-Delay: Decision screen

Please carefully consider your decision.

You must think for more than 10 seconds before submitting your decision.

How many tokens do you wish to contribute to the group project?

Your contribution:

0 10

Maintenance – No Time-Limit: Preliminary Screen

Part A

Part A will now begin.

Please continue.

Maintenance – No Time-Limit: Decision Screen

How many tokens do you wish to withdraw from the group project?

Your withdrawal:

0 10

Maintenance – Time-Pressure: Preliminary Screen (displayed for 15s)

Part A

Please continue reading now.

The next screen will appear very soon.

On the next screen, you will need to submit your decision in less than 10 seconds in order to be eligible for any payments.

If you take more than 10 seconds to submit your decision, then you will not get paid.

Maintenance – Time-Pressure: Decision Screen (Note: Study 2 has a 5 second time-limit)

Please be quick. You must submit your decision in less than 10 seconds!

How many tokens do you wish to withdraw from the group project?

Your withdrawal:

0 10

Maintenance – Time-Delay: Preliminary Screen (displayed for 15s)

Part A

Please continue reading now.

The next screen will appear very soon.

On the next screen, you will need to think for more than 10 seconds before submitting your decision in order to be eligible for any payments.

If you submit your decision in less than 10 seconds, then you will not get paid.

Maintenance – Time-Delay: Decision Screen

Please carefully consider your decision.

You must think for more than 10 seconds before submitting your decision.

How many tokens do you wish to withdraw from the group project?

Your withdrawal:

0 10

Part B: Expectations

Provision – No Time-Limit: Preliminary Screen

Part B

You have competed Part A. Please continue reading.

You will next be asked to make an estimation (rounded to the nearest whole number).

The question has a correct answer. You will receive 50 pence if your estimate is correct.

If your estimate is wrong, then you will not get paid for this question.

Provision – No Time-Limit: Decision Screen

What is the average number of tokens contributed by the other people in your group?

0 10

Provision – Time-Pressure: Preliminary Screen (displayed for 20s)

Part B

You have competed Part A.

Please continue reading now.

You will next be asked to make an estimation (rounded to the nearest whole number).

The question will appear very soon.

The question has a correct answer. You will receive 50 pence if your estimate is correct and if you answer in less than 10 seconds.

If your estimate is wrong or if you answer in more than 10 seconds, then you will not get paid for this question.

Provision – Time-Pressure: Decision Screen (Note: Study 2 has a 5 second time-limit)

Please be quick. You must submit your decision in less than 10 seconds!

What is the average number of tokens contributed by the other people in your group?

0 10

Provision – Time-Delay: Preliminary Screen (displayed for 20s)

Part B

You have competed Part A.

Please continue reading now.

You will next be asked to make an estimation (rounded to the nearest whole number).

The question will appear very soon.

The question has a correct answer. You will receive 50 pence if your estimate is correct and if you answer after thinking for more than 10 seconds.

If your estimate is wrong or if you submit your answer in less than 10 seconds, then you will not get paid for this question.

Provision – Time-Delay: Decision Screen

Please carefully consider your decision.

You must think for more than 10 seconds before submitting your decision.

What is the average number of tokens contributed by the other people in your group?

0 10

Maintenance – No Time-Limit: Preliminary Screen

Part B

You have competed Part A. Please continue reading.

You will next be asked to make an estimation (rounded to the nearest whole number).

The question has a correct answer. You will receive 50 pence if your estimate is correct.

If your estimate is wrong, then you will not get paid for this question.

Maintenance – No Time-Limit: Decision Screen

What is the average number of tokens withdrawn by the other people in your group?

0 10

Maintenance – Time-Pressure: Preliminary Screen (displayed for 20s)

Part B

You have competed Part A.

Please continue reading now.

You will next be asked to make an estimation (rounded to the nearest whole number).

The question will appear very soon.

The question has a correct answer. You will receive 50 pence if your estimate is correct and if you answer in less than 10 seconds.

If your estimate is wrong or if you answer in more than 10 seconds, then you will not get paid for this question.

Maintenance – Time-Pressure: Decision Screen (Note: Study 2 has a 5 second time-limit)

Please be quick. You must submit your decision in less than 10 seconds!

What is the average number of tokens withdrawn by the other people in your group?

0 10

Maintenance – Time-Delay: Preliminary Screen (displayed for 20s)

Part B

You have competed Part A.

Please continue reading now.

You will next be asked to make an estimation (rounded to the nearest whole number).

The question will appear very soon.

The question has a correct answer. You will receive 50 pence if your estimate is correct and if you answer after thinking for more than 10 seconds.

If your estimate is wrong or if you submit your answer in less than 10 seconds, then you will not get paid for this question.

Maintenance – Time-Delay: Decision Screen

Please carefully consider your decision.

You must think for more than 10 seconds before submitting your decision.

What is the average number of tokens withdrawn by the other people in your group?

0 10

Part B: Understanding

No Time-Limit Conditions: Preliminary Screen

You are still in Part B.

You will next be answering two questions on the same screen.

Each question has a correct answer. For each question you will receive 50 pence if your answer is correct.

If your answer is wrong, then you will not get paid for that question.

Time-Limit Conditions: Preliminary Screen

You are still in Part B.

You will next be answering two questions on the same screen **at your own pace**.

Each question has a correct answer. For each question you will receive 50 pence <u>if your answer is correct</u>.

If your answer is wrong, then you will not get paid for that question.

Provision: Decision Screen [Note: question order is randomized]

Please carefully read both questions first before you answer each question.

0 10

What level of contribution by you (in tokens) earns <u>the most money for you personally</u>?

What level of contribution by you (in tokens) earns <u>the most money for the group as a whole</u>?

Maintenance: Decision Screen [Note: question order is randomized]

Please carefully read both questions first before you answer each question.

0 10

What level of withdrawal by you (in tokens) earns <u>the most money for the group as a whole</u>?

What level of withdrawal by you (in tokens) earns <u>the most money for you personally</u>?

Part C: Cooperation Preferences

Provision: Decision Screen (Note: single screen with randomized question order)

Part C

You and the other three people from Part A will interact again as a group in Part C. Tasks in Part A and Part C are the same with one exception: The level of contribution to the project by the other people in your group will be exactly the same in Part C as it was in Part A.

In Part C, you must decide how many of 10 tokens to contribute to the group project for each possible average level of contribution that the other people in your group could have made in Part A. The outcome of Part C depends on what you will choose in Part C and what the other people in your group have actually chosen in Part A.

The relationship between contributions to the group project and earnings is exactly the same as in Part A, which you can review at the bottom of the screen if you want to.

Each possible average level of contribution by the other people in your group is stated on the left side of the sliders (rounded to the nearest whole number). Using the sliders below, please fill in the "Contribution Table" by indicating how many tokens you want to contribute to the project for each possible average contribution level of the other people in your group. Choices you make on a slider will be shown on the right side of the slider. Once you have made an entry for all eleven sliders, click the "SUBMIT" button at the end of the screen.

Contribution Table

How many tokens do you want to contribute to the group project if the other people in your group have on average contributed...

	0 — 10
**0 tokens**	
**1 token**	
**2 tokens**	
**3 tokens**	
**4 tokens**	
**5 tokens**	
**6 tokens**	
**7 tokens**	
**8 tokens**	
**9 tokens**	
**10 tokens**	

Review

At the end of the interaction, each 10 pence token contributed to the group project will increase the value of the project by 20 pence, which will then be split evenly between the four of you. Thus, for every token contributed to the group project, each group member receives 5 pence.

For example, if everyone keeps 10 tokens for oneself, then the value of the project would be zero and total earnings from the interaction for each person would be 100 pence.

If everyone contributes 10 tokens to the project, then the value of the project would be 800 pence and each of you will earn 200 pence in total.

But if everyone else contributes 10 tokens to the project, while you keep 10 tokens for yourself, you will earn 250 pence, while the others will earn only 150 pence. That is because for every token you contribute to the project, you get only 5 pence back.

Maintenance: Decision Screen (Note: single screen with randomized question order)

Part C

You and the other three people from Part A will interact again as a group in Part C. Tasks in Part A and Part C are the same with one exception: The level of withdrawal from the project by the other people in your group will be exactly the same in Part C as it was in Part A.

In Part C, you must decide how many tokens, up to a maximum of 10, to withdraw from the group project for each possible level of withdrawal that the other people in your group could have made in Part A. The outcome of Part C depends on what you will choose in Part C and what the other people in your group have actually chosen in Part A.

The relationship between withdrawals from the group project and earnings is exactly the same as in Part A, which you can review at the bottom of the screen if you want to.

Each possible average level of withdrawal by the other people in your group is stated on the left side of the sliders (rounded to the nearest whole number). Using the sliders below, please fill in the "Withdrawal Table" by indicating how many tokens you want to withdraw from the project for each possible average withdrawal level of the other people in your group. Choices you make on a slider will be shown on the right side of the slider. Once you have made an entry using all eleven sliders, click the "SUBMIT" button at the end of the screen.

<u>Withdrawal Table</u>

How many tokens do you want to withdraw from the group project if the other people in your group have on average withdrawn...

	0 — 10
**10 tokens**	
**9 tokens**	
**8 tokens**	
**7 tokens**	
**6 tokens**	
**5 tokens**	
**4 tokens**	
**3 tokens**	
**2 tokens**	
**1 token**	
**0 tokens**	

<u>Review</u>

At the end of the interaction, each 10 pence token left in the group project will increase the value of the project by 20 pence, which will then be split evenly between the four of you. Thus, for every token left in the group project, each group member receives 5 pence.

For example, if everyone leaves 10 tokens in the project, then the value of the project would be 800 pence and total earnings from the interaction for each person would be 200 pence.

If everyone withdraws 10 tokens for themselves, then the value of the project would be zero and each of you will earn 100 pence in total.

But if everyone else leaves 10 tokens in the project, while you withdraw 10 tokens for yourself, you will earn 250 pence, while the others will earn only 150 pence. That is because for every token you leave in the project, you get only 5 pence back.

Part D: Cognitive Reflection Task (Study 2 only)

Part D

You will next be answering three questions **at your own pace**.

You will receive 50 pence for completing Part D.

Q1

A bat and a ball cost £1.10 in total. The bat costs £1.00 more than the ball. How much does the ball cost?

[ ] pence

Q2

If it takes 5 machines 5 minutes to make 5 widgets, how long would it take 100 machines to make 100 widgets?

[ ] minutes

Q3

In a lake, there is a patch of lily pads. Every day, the patch doubles in size. If it takes 48 days for the patch to cover the entire lake, how long would it take for the patch to cover half of the lake?

[ ] days

5. Supplementary References

1. Frederick S (2005) Cognitive reflection and decision making. *Journal of Economic Perspectives* 19(4):24-42.
2. Cueva C*, et al.* (2016) Cognitive (ir)reflection: New experimental evidence. *Journal of Behavioral and Experimental Economics* 64:81-93.
3. Stagnaro MN, Pennycook G, Rand DG (2018) Performance on the cognitive reflection test is stable across time. *Judgment and Decision Making* 13(3):260-267.
4. Bialek M, Pennycook G (2018) The cognitive reflection test is robust to multiple exposures. *Behav Res* 50(5):1953-1959.
5. Chandler J, Mueller P, Paolacci G (2014) Nonnaïveté among amazon mechanical turk workers: Consequences and solutions for behavioral researchers. *Behav Res* 46(1):112-130.
6. Krajbich I, Bartling B, Hare T, Fehr E (2015) Rethinking fast and slow based on a critique of reaction-time reverse inference. *Nat Commun* 6.
7. Spiliopoulos L, Ortmann A (2018) The bcd of response time analysis in experimental economics. *Experimental Economics* 21(2):383-433.
8. Ratcliff R, McKoon G (2008) The diffusion decision model: Theory and data for two-choice decision tasks. *Neural Computation* 20(4):873-922.
9. Recalde MP, Riedl A, Vesterlund L (2018) Error-prone inference from response time: The case of intuitive generosity in public-good games. *Journal of Public Economics* 160:132-147.
10. Merkel AL, Lohse J (2019) Is fairness intuitive? An experiment accounting for subjective utility differences under time pressure. *Experimental Economics* 22(1):24-50.
11. Chen F, Krajbich I (2018) Biased sequential sampling underlies the effects of time pressure and delay in social decision making. *Nature Communications* 9(1):3557.
12. Evans AM, Dillon KD, Rand DG (2015) Fast but not intuitive, slow but not reflective: Decision conflict drives reaction times in social dilemmas. *J. Exp. Psychol.-Gen.* 144(5):951-966.
13. Isler O, Maule J, Starmer C (2018) Is intuition really cooperative? Improved tests support the social heuristics hypothesis. *PLOS ONE* 13(1):e0190560.
14. Mischkowski D, Glöckner A (2016) Spontaneous cooperation for prosocials, but not for proselfs: Social value orientation moderates spontaneous cooperation behavior. *Scientific Reports* 6(1):21555.
